# Quantitative metaproteomics reveals composition and metabolism characteristics of microbial communities in Chinese liquor fermentation starters

**DOI:** 10.3389/fmicb.2022.1098268

**Published:** 2023-01-09

**Authors:** Jinzhi Zhao, Yi Yang, Liangqiang Chen, Jianxujie Zheng, Xibin Lv, Dandan Li, Ziyu Fang, Chengpin Shen, Vijini Mallawaarachchi, Yu Lin, Shaoning Yu, Fan Yang, Li Wang, Liang Qiao

**Affiliations:** ^1^Department of Chemistry and Shanghai Stomatological Hospital, Fudan University, Shanghai, China; ^2^Kweichow Moutai Group, Renhuai, Guizhou, China; ^3^Department of Chemistry and Chemical Biology, Rensselaer Polytechnic Institute, Troy, NY, United States; ^4^Shanghai Omicsolution Co., Ltd., Shanghai, China; ^5^College of Engineering and Computer Science, The Australian National University, Canberra, ACT, Australia; ^6^Zhejiang Provincial Key Laboratory of Advanced Mass Spectrometry and Molecular Analysis, Institute of Mass Spectrometry, School of Material Science and Chemical Engineering, Ningbo University, Ningbo, China

**Keywords:** *Daqu*, Chinese liquor, microbial community, quantitative metaproteomics, data-independent acquisition

## Abstract

**Introduction:**

*Daqu*, the Chinese liquor fermentation starter, contains complex microbial communities that are important for the yield, quality, and unique flavor of produced liquor. However, the composition and metabolism of microbial communities in the different types of high-temperature *Daqu* (i.e., white, yellow, and black *Daqu*) have not been well understood.

**Methods:**

Herein, we used quantitative metaproteomics based on data-independent acquisition (DIA) mass spectrometry to analyze a total of 90 samples of white, yellow, and black *Daqu* collected in spring, summer, and autumn, revealing the taxonomic and metabolic profiles of different types of *Daqu* across seasons.

**Results:**

Taxonomic composition differences were explored across types of *Daqu* and seasons, where the under-fermented white *Daqu* showed the higher microbial diversity and seasonal stability. It was demonstrated that yellow *Daqu* had higher abundance of saccharifying enzymes for raw material degradation. In addition, considerable seasonal variation of microbial protein abundance was discovered in the over-fermented black *Daqu*, suggesting elevated carbohydrate and amino acid metabolism in autumn black *Daqu*.

**Discussion:**

We expect that this study will facilitate the understanding of the key microbes and their metabolism in the traditional fermentation process of Chinese liquor production.

## 1. Introduction

*Daqu* is the fermentation starter for brewing Chinese liquor (*Baijiu*; Zheng et al., 2011). It is produced from carbohydrate-rich raw materials (e.g., barley, wheat, and peas) and aerobically manufactured through a spontaneous solid-state fermentation with inoculation of indigenous microorganisms in the environment (Wu et al., 2016; Yang et al., 2021). High-temperature *Daqu* is usually used as the fermentation starter for sauce-flavore *Baijiu* production (Wang et al., 2021). Factors like the fermentation temperature varying at different positions in the starter brick result in three types of *Daqu* with different colors after fermentation, i.e., white *Daqu* (under-fermented starters), yellow *Daqu* (well fermented starters), and black *Daqu* (over-fermented starters; Gan et al., 2019; Wang et al., 2021). In hot and humid fermentation environment, the Maillard reaction of amino acids and sugars produces brown compounds, which appears to give *Daqu* its color (Zheng et al., 2011). In actual production, the three types of *Daqu* are mixed mainly depends on the workers’ experience. The fermentation temperature can potentially contribute to the formation of different microbial communities of *Daqu*. The fermentation yield and quality are closely related to the microorganisms involved in the brewing process (Yang et al., 2018; Wang et al., 2021), and the special microbial compositions in *Daqu* can contribute to the unique aromas and tastes of the produced liquor (Gan et al., 2019). In 2014, Li et al. analyzed the connection between the flavor components of Baijiu and the dominant flavor-producing bacteria isolated from the central black component of *Daqu* (Li et al., 2014). Since then, many studies have reported differences of microbial community structure among the three types of *Daqu* (Gan et al., 2019; Deng et al., 2020; Wang et al., 2021). More recently, Cai et al. reported that the fungal communities in the three types of *Daqu* were significantly correlated with taste of *Baijiu* by high-throughput sequencing and electronic senses (Cai et al., 2021). Hence, it is important to clarify the microbial community structure in different types of *Daqu* and their function in liquor brewing to improve the product quality of *Baijiu*.

Various methods have been applied to explore the microbial composition in *Daqu*. The classical microbiological methods based on bacterial culture or PCR-denaturing gradient gel electrophoresis have identified many common microbes in the *Baijiu* fermentation (Zheng et al., 2011; Lv et al., 2012; Wang et al., 2016). Metagenomics based on high-throughput sequencing has enabled the discovery of the unculturable and low-abundant microbes, revealing the composition and diversity of *Daqu* microbial communities in breadth and depth (Su et al., 2015; Hu et al., 2017; Gan et al., 2019; Wang et al., 2019, 2021). However, knowledge of community structure characterized solely by metagenomics does not necessarily provide useful information on functions such as metabolic capacity and population dynamics (Wu et al., 2016).

As a powerful mean providing information about protein composition and relevant metabolic pathways, metaproteomics has been applied to investigate enzymatic proteins of microbes in *Baijiu* production (Wu et al., 2016; Wang et al., 2018, 2020; Xia et al., 2022). However, few studies have investigated the metaproteome of microbial communities in *Daqu* with large sample size. Recently, data-independent acquisition (DIA) mass spectrometry has shown the applicability to the analysis of complex metaproteomic samples (Aakko et al., 2020; Long et al., 2020; Pietilä et al., 2022), circumventing the drawbacks of the previously used data-dependent acquisition (DDA) method wherein the stochastic precursor ion selection leads to low reproducibility (Tabb et al., 2010). Moreover, the latest library-free DIA data analysis methods (Muntel et al., 2019; Gotti et al., 2021; Pietilä et al., 2022), such as directDIA (Muntel et al., 2019), no longer require additional DDA data to assist the protein identification and need only a single-shot DIA analysis per sample, enabling identification and quantification of proteins in large sample cohorts.

Herein, we used DIA-based quantitative metaproteomics to analyze a total of 90 samples of white, yellow, and black *Daqu* produced in spring, summer, and autumn. Composition differences of microbial taxa and saccharifying enzymatic proteins were revealed across types of *Daqu* and seasons. Moreover, the functional characteristics of the microbial proteins in different seasons were explored, revealing the varied metabolic features of *Daqu* microbiota across seasons. We expect that this study will aid in uncovering the key microbes and their metabolism in the fermentation process of *Baijiu* and facilitate the deeper understanding of this traditional technique.

## 2. Materials and methods

### 2.1. Sample collection

The *Daqu* samples was collected from Kweichow Moutai Liquor Co., Ltd. (Guizhou, China). Ninety samples were collected from *Daqu* finished products in spring, summer, and autumn, including 10 pieces of white, yellow, and black *Daqu* in each season. All *Daqu* samples were produced by the traditional high-temperature *Daqu* production method (Wang et al., 2021). The collected samples were stored in dry ice and transported back to the laboratory within 24 h for storage at −80°C.

### 2.2. DNA extraction, sequencing, and assembly

For each of the 90 *Daqu* samples, 0.2 g were weighed and then combined into 9 samples according to the seasons and types. The DNA of the pooled samples was extracted using HiPure Bacterial DNA Kits (Magen, Guangzhou, China) according to the manufacturer’s instructions. The DNA quality was detected using Qubit and Nanodrop (Thermo Fisher Scientific, MA, United States).

Qualified genomic DNA was firstly fragmented by sonication to a size of 350 bp, and then end-repaired, A-tailed, and adaptor ligated using NEBNext® ΜLtra™ DNA Library Prep Kit for Illumina (NEB, MA, United States) according to the preparation protocol. DNA fragments with length of 300–400 bp were enriched by PCR and then purified using AMPure XP system (Beckman Coulter, CA, United States). The libraries were analyzed for size distribution by 2,100 Bioanalyzer (Agilent, CA, United States) and quantified using real-time PCR. Genome sequencing was performed on a NovaSeq 6,000 sequencer (Illumina Inc., CA, United States) using pair-end technology (PE 150).

Raw data from Illumina platform was filtered using FASTP (version 0.18.0; Chen et al., 2018) by the following standards: (1) removing reads with ≥10% unidentified nucleotides (N); (2) removing reads with ≥50% bases having Phred quality scores ≤ 20; (3) removing reads aligned to the barcode adapter. After filtering, resulted clean reads were used for genome assembly. Clean reads of each sample were assembled individually using MEGAHIT (version 1.2.9; Li et al., 2015). Genes were identified using MetaGeneMark (version 3.38; Tang and Borodovsky, 2013). The output amino acid sequences of the genes were then used as the protein database for metaproteomics analysis.

### 2.3. Sample preparation for metaproteomics

For each individual *Daqu* sample, 0.5 g was treated with a mechanical grinder (JXFSTPRP-CL, Shanghai Jingxin Industrial Development Co., Ltd., Shanghai, China). After grinding twice with 5 steel balls (−50°C, 70 Hz, on 120 s, off 120 s), 1 ml BPP solution (100 mM EDTA, 50 mM borax, 50 mM vitamin C, 30% sucrose, 10 mM Tris-base, 1% Triton-100, 5 mM 1,4-dithiothreitol, 1% polyvinylpolypyrrolidone) was added to the sample. After vortexing, 1 ml DNA extraction phenol reagent (Beijing Solarbio Science & Technology Co., Ltd., Beijing, China) was added. The sample was vortexed (2 min) and centrifuged (12,000 *g*, 20 min, 4°C). The upper phenol phase was taken. Another 1 ml of BPP solution was added to the phenol phase, and the lysis step was repeated once. Then 5 ml of methanol solution containing 0.1 M ammonium acetate pre-cooled at −20°C was added to the upper phenol phase, and the sample was precipitated at −20°C for 4 h. The sample was centrifuged (12,000 *g*, 20 min, 4°C) and the supernatant was discarded. The precipitate was washed with 1 ml of 0.1 M ammonium acetate methanol solution twice. After drying at room temperature, 300 μl of 1% sodium dodecyl sulfate (SDS) solution with 8 M urea was added to dissolve the protein.

Different lysates and cell disruption methods were also benchmarked using 0.5 g summer yellow *Daqu* sample. (1) Sample 1 was mechanical grinded twice with 5 steel balls. Then 1 ml 1% sodium deoxycholate (SDC) lysate containing 1X protease inhibitor cocktail (Thermo Fisher Scientific, MA, United States) was added. After grinding twice, the supernatant solution was obtained by centrifugation. (2) Sample 2 was mechanical grinded same as Sample 1, and treated with 1 ml 1% SDS lysate containing 8 M urea and 1X protease inhibitor cocktail. (3) Sample 3 was treated with mechanical grinding same as Sample 1 and BPP lysate as described above. (4) Sampel 4 was grinded with liquid nitrogen, and then treated with BPP lysate same as Sample 3.

The protein was quantified using Pierce BCA protein assay kit (Thermo Fisher Scientific, MA, United States). For each sample, 8 M urea solution and 1 M Triethylammonium bicarbonate (TEAB) buffer solution was added to 200 μg protein to a volume of 200 μl (the final concentration of TEAB was 0.1 M). Then 4 μl 0.5 M tris-(2-carboxyethyl) phosphine hydrochloride (TCEP) was added. After shaking (600 rpm, 1 h, 37°C), 18 μl 0.5 M iodoacetamide was added and the sample was reacted for 45 min in the dark at room temperature. Then 1.2 ml acetone pre-cooled at −20°C was added, and the sample was precipitated at −20°C for 4 h. After high-speed centrifugation (15,000 g, 15 min, 4°C) to remove the supernatant, the precipitate was washed twice with 90% acetone, dried at room temperature, and fully dispersed in 200 μl 0.1 M TEAB solution. The protein was digested (600 rpm, 16 h, 37°C) with 4 μg trypsin (Beijing Wallis Technology Co., Ltd., Beijing, China). The MonoSpin C18 column (Shimadzu, Tokyo, Japan) was used for desalting, and the Pierce quantitative colorimetric peptide assay kit (Thermo Fisher Scientific, MA, United States) was used for peptide quantification.

### 2.4. LC–MS/MS analysis

All samples were analyzed by an online nanospray Orbitrap Fusion Lumos Tribrid mass spectrometer (Thermo Fisher Scientific, MA, United States) coupled with a Nano ACQUITY UPLC system (Waters Corporation, MA, United States). The peptides (10 μg) were resuspended in 30 μl solvent A (0.1% formic acid in water) spiked with 1X iRT peptides (Biognosys AG, Schlieren, Switzerland). Then 1 μg peptides were loaded to an C18 column (Acclaim PepMap, 75 μm × 25 cm, Thermo Fisher Scientific, MA, United States) and separated with a gradient of 60 min (Supplementary Table 1), from 2 to 95% solvent B (0.1% formic acid, 20% water and 80% acetonitrile). The flow rate was 250 nl/min and the column temperature was 40°C.

The 90 individual *Daqu* samples were analyzed by DIA mode LC–MS/MS. The parameters were (1) MS1: resolution = 120,000; scan range (*m*/*z*) = 349.5–1500.5; AGC target = 4e5; (2) MS/MS: resolution = 30,000; AGC target = 50,000; maximum injection time = 30 ms; collision energy = 32%. Sixty variable windows were set for MS2 acquisition (Supplementary Table 2).

The samples for benchmarking different lysates and cell disruption methods were analyzed by DDA mode LC–MS/MS. The parameters were (1) MS1: resolution = 15,000; scan range (m/z) = 350–1,800; (2) MS/MS: resolution = 15,000; collision energy = 30%. Other parameters were the same as those for DIA.

### 2.5. MS data analysis

The DIA raw data were analyzed by Spectronaut (Bruderer et al., 2015; version 15.4.210913, Biognosys AG, Schlieren, Switzerland). The data were searched by directDIA against the protein sequence database built from metagenomic sequencing (1,614,586 protein entries) without the need of spectral libraries. Trypsin was used for proteolysis and the maximum number of missed cleavages was 2. Q-value cut-off at both precursor and protein level was set as 1%. Missing value imputation was set to global. Other parameters were default. To save computing resources, raw data of each *Daqu* type (30 files each) were searched in a separate project, and the results were merged using the SNE Combine function in Spectronaut.

The DDA raw data were analyzed by PEAKS Studio (Lin et al., 2013; version X+, Bioinformatics Solutions Inc., Waterloo, Canada). The mass tolerance of precursor and fragment ion was set as 7 ppm and 0.02 Da, respectively. Carbamidomethylation (C) was set as a fixed modification, while Oxidation (M) and acetylation (K) were specified as variable modifications. The false discovery rate (FDR) cut-off on both peptide and protein level was 1% by using a target-decoy strategy. Proteins were identified based on at least one unique peptide. Other parameters were default.

Protein inference was performed by the software for protein identification and quantification. Only the leading protein (with the strongest evidence and ranked first in the result) in each protein group was taken into consideration in all the subsequent analysis.

### 2.6. Statistical analysis

Partial least squares discriminant analysis was performed on the protein quantified results using MetaboAnalyst (Pang et al., 2022; version 5.0, https://www.metaboanalyst.ca/, accessed in December 2022). Proteins with >50% missing values were removed and missing values were replaced by 1/5 of min positive values of their corresponding variables. Normalization by sum, log transformation, and auto scaling were conducted. The predictive ability of the model was measured *via* leave-one-out cross-validation.

### 2.7. Taxonomic analysis

The quantified peptides were subjected to Unipept (Gurdeep Singh et al., 2019; version 4.3, https://unipept.ugent.be/, accessed in May 2022) for taxonomic analysis using the lowest common ancestor approach. Leucine and isoleucine were considered equal. If a taxon has only one peptide, it was removed and the peptide was assigned to the parental taxon. Abundance of each taxon was determined by summing the quantities of all peptides corresponding to the taxon, which was then converted to percentages to the total abundance of the “root” taxon. Taxonomic abundance differences across seasons were determined by Kruskal-Wallis test (*p*-value < 0.01) followed by pairwise Mann–Whitney U test (*p*-value < 0.01). In an alternative strategy, the abundance of taxon was determined by the number of identified peptides of the taxon.

Co-occurrence patterns of the genera were analyzed by Spearman’s rank correlations. Genera with relative abundance >0.05% and significant correlation (Spearman’s correlation > 0.8, *p*-value < 0.05 with Bonferroni adjustment) were taken into consideration. The igraph R package (version 1.2.7, https://igraph.org/r/) was used to build the correlation networks. Nodes were divided into clusters (densely connected subgraphs) by the Walktrap community finding algorithm (Pons and Latapy, 2005).

### 2.8. Protein functional analysis

The quantified proteins were annotated with eggNOG (Huerta-Cepas et al., 2019; version 5.0, http://eggnogdb.embl.de/, accessed in May 2022). COG and KO annotations, as well as EC number and taxonomic information were extracted from the eggNOG results.

Differential proteins across seasons were determined by fold change (FC > 2 or FC < 0.5) and Kruskal-Wallis test (adjusted *p*-value < 0.05) followed by pairwise Mann–Whitney U test (adjusted *p*-value < 0.05) with the Benjamini-Hochberg adjustment. For each of the dominant taxa assigned to the differential proteins, KEGG enrichment analysis was perform using the clusterProfiler R package (Wu et al., 2021; version 4.2.0, https://github.com/YuLab-SMU/clusterProfiler). The KO numbers matched with the differential proteins were used as the gene list of interest. The KEGG entries of all the organisms that belong to the taxon and are available in the KEGG database (Kanehisa et al., 2017; https://www.genome.jp/kegg/, accessed in May 2022) were used as the background genes. The *p*-values were adjusted using the Benjamini-Hochberg method.

### 2.9. Data processing and visualization

Data processing and statistics were performed using Python (version 3.9.7, Anaconda distribution version 2021.11, https://www.anaconda.com/) and R (version 4.0.2, Microsoft R Open distribution, https://mran.microsoft.com/open). Gephi (version 0.9.5, https://gephi.org/) was used for network visualization. AntV G2 (version 3.2.7, https://g2.antv.vision/) was used to plot the cladograms illustrating abundance of taxa. The volcano3D R package (Kobayashi et al., 2019; version 1.3.2, https://github.com/KatrionaGoldmann/volcano3D) was used to generate the polar and volcano plots of the differential proteins. The R packages ggplot2 (version 3.3.5), ggpubr (version 0.4.0), and VennDiagram (version 1.6.20) were used for other data visualization.

## 3. Results and discussion

### 3.1. Metaproteome profiles of white, yellow, and black *Daqu*

**Figure 1:**
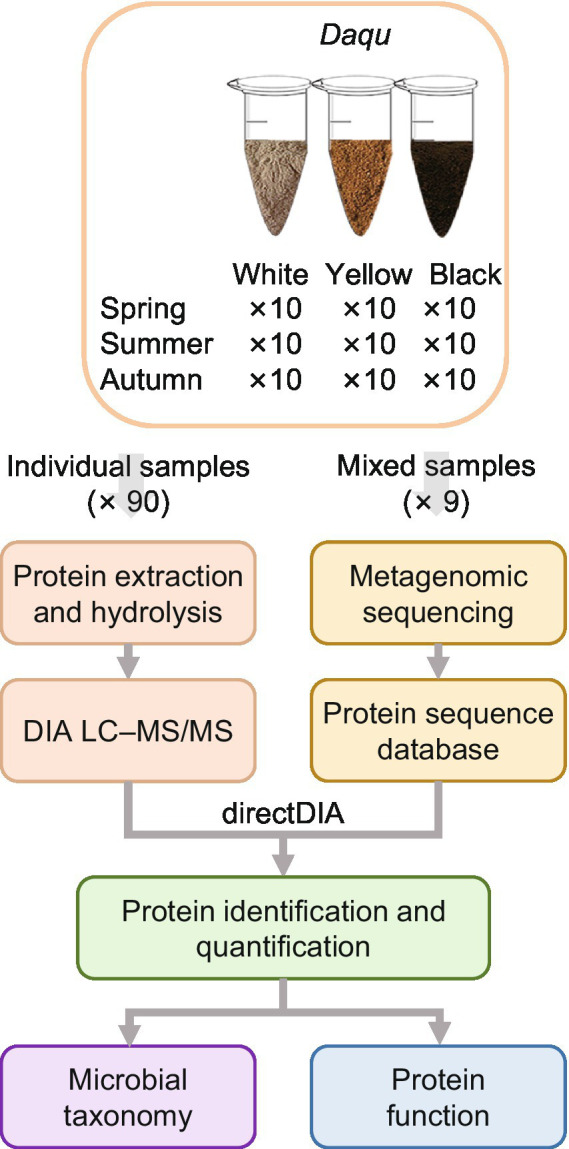
Workflow of quantitative metaproteomics analysis of *Daqu* in this study. Metagenomics sequencing was performed on the pooled samples to build the protein sequence database for metaproteomics analysis. The individual samples were characterized by metaproteomics using directDIA.

**Figure 2:**
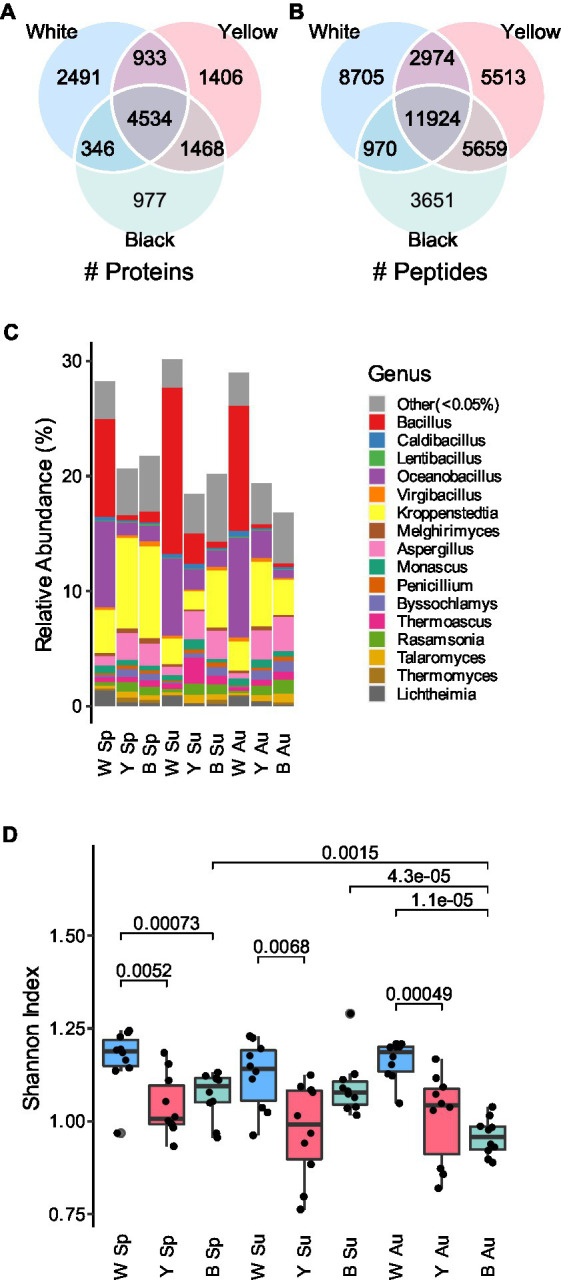
Overview of the metaproteomics analysis results of the *Daqu* samples. **(A)** Overlap of the proteins detected in white, yellow, and black *Daqu*. **(B)** Overlap of the peptides detected in the three types of *Daqu*. **(C)** Relative abundances of genera base on peptide quantities in the *Daqu* microbiota. **(D)** Alpha diversity at the genus level. The boxes mark the first and third quantile and the lines inside the boxes mark the median; the whiskers extend from the ends of the inter-quartile range (IQR) to the furthest observations within the 1.5 times the IQR. Individual data points are overlaid as dots. The *p*-values are indicated if they are <0.05. W: white; Y: yellow; B: black; Sp: spring season; Su: summer season; Au: autumn season.

We collected 10 samples in each season (i.e., spring, summer, and autumn) for each type of *Daqu* (i.e., white, yellow, and black *Daqu*), bringing the total to 90 samples (Figure 1). The making process of *Daqu* and the sampling time is illustrated in Supplementary Figure 1. To build a protein sequence database for metaproteomics analysis, the 10 samples of each season and type were mixed into a pooled sample (i.e., 9 pooled samples in total), which was subjected to metagenomics sequencing. The assembled genomes were translated to amino acid sequences to form a sample-specific protein sequence database (1,614,586 protein entries). It is a challenge to extract proteins from *Daqu* due to the existence of complicated matrices of raw materials. We compared different protein extraction methods. The borax/polyvinylpolypyrrolidone/phenol (BPP) lysate (Wang et al., 2007) and mechanical grinding showed the best performance (Supplementary Note 1; Supplementary Figure 2). Then, the 90 individual samples were treated with the optimized protein extraction method, and analyzed by DIA-based liquid chromatography–tandem mass spectrometry (LC–MS/MS). The DIA data were searched against the sample-specific database using directDIA, resulting in 39,396 peptides of 12,155 proteins identified and quantified in total (Figures 2A,B). The three types of *Daqu* shared 37% of the total proteins. White *Daqu* contained the largest number of proteins (8,304, 68%), while black *Daqu* contained the least number of proteins (7,325, 60%). At the peptide level, the trend of identification numbers across *Daqu* types was consistent with that at the protein level. For each season, PLS-DA was performed using the proteins quantified in ≥50% of the samples. The PLS-DA score plots showed obvious separations among the three types of *Daqu* using the first three components (Supplementary Figure 3). *Q*^2^ was >80% for samples in spring and autumn and slightly lower in summer using the first three components, indicating good predictive ability of the PLS-DA models to distinguish the three types of *Daqu*.

We further investigated the taxonomy features contributing to the differentiation of the three types of *Daqu*. Taxonomic information was assigned to the quantified peptides using Unipept (Gurdeep Singh et al., 2019). Among the 23,070 annotated peptides, 12,462 were matched to 93 classes, and 7,539 were matched to 449 genera. Relative abundances of taxa were measured by summarizing the (1) intensities or (2) counts of the corresponding peptides annotated to each taxon (Supplementary Data 1), and expressed as percentages to all the annotated peptides (Supplementary Figures 4, 5). Similar results were obtained using the two quantification strategies. The ratio of fungi to bacteria in white *Daqu* was lower than that in yellow and black *Daqu*. White *Daqu* contains more abundant Bacilli, mainly consisting of *Bacillus* and *Oceanobacillus*, than yellow and black *Daqu*. Conversely, Eurotiomycetes, including *Kroppenstedtia* and *Aspergillus*, were less abundant in white *Daqu* (Figure 2C). We further measured the alpha diversity of the genera in *Daqu* microbial communities. The Shannon indexes of white *Daqu* were significantly higher than those of yellow and black *Daqu* in each season (Figure 2D; Supplementary Figure 6). This result was inconsistent with the previous metagenomics study that no significant difference of microbial diversity was observed among the three types of *Daqu* (Wang et al., 2021). It is reasonable that taxonomic abundances based on metagenomics and metaproteomics are different because of divergences between genetic potential and expressed functional activity (Tanca et al., 2017). The quantification based on metagenomics is closely related to the relative cell number, while the quantification based on metaproteomics is closely related to the protein amount of a taxon. The elevated proportion of fungi and the decreased taxonomic diversity in yellow and black *Daqu* is probably a result of the higher fermentation level.

We then explored the co-occurrence patterns of *Daqu* microbial communities based on Spearman’s rank correlations (Supplementary Data 2). In the microbial communities of black *Daqu*, 217 significant correlations (edges) were identified from 55 genera (nodes) with an average degree of 7.9 (Supplementary Figure 7). Genera in the Bacillaceae family (e.g., *Oceanobacillus* and *Weizmannia*) exhibited positive correlations. The lactic acid bacteria *Weissella* were positively related with the occurrence of a range of filamentous fungi. Co-occurrence networks of white (188 edges among 44 nodes with an average degree of 8.5) and yellow *Daqu* (217 edges among 58 nodes with an average degree of 8.0) are also shown in Supplementary Figures 8, 9. The largest module of co-occurrence network in white *Daqu* consisted of 22 fungal genera including *Aspergillus* and *Thermoascus*, while that of yellow *Daqu* contained 16 nodes mostly composed of genera in the Bacillaceae family. Despite less nodes and edges, higher average degree in the co-occurrence network of white *Daqu* than those in yellow and black *Daqu*, suggesting closer interconnections and more frequent co-occurrence among microbial taxa in white *Daqu*. White Daqu in the top layer of the starter brick is more sufficiently exposed to air. In contrast, black *Daqu* is distributed in the central part of the starter brick with high temperature and low oxygen tension, inhibiting the growth of non-thermophilic or aerobic microorganisms.

### 3.2. Taxonomic composition of *Daqu* microbiome across seasons

We next compared the taxonomic abundance of *Daqu* microbial communities in the three different seasons. While no significant change of the Shannon indexes among the three seasons was observed for white and yellow *Daqu*, microbial communities of black *Daqu* presented lower diversity in autumn than the other seasons (Figure 2D).

**Figure 3:**
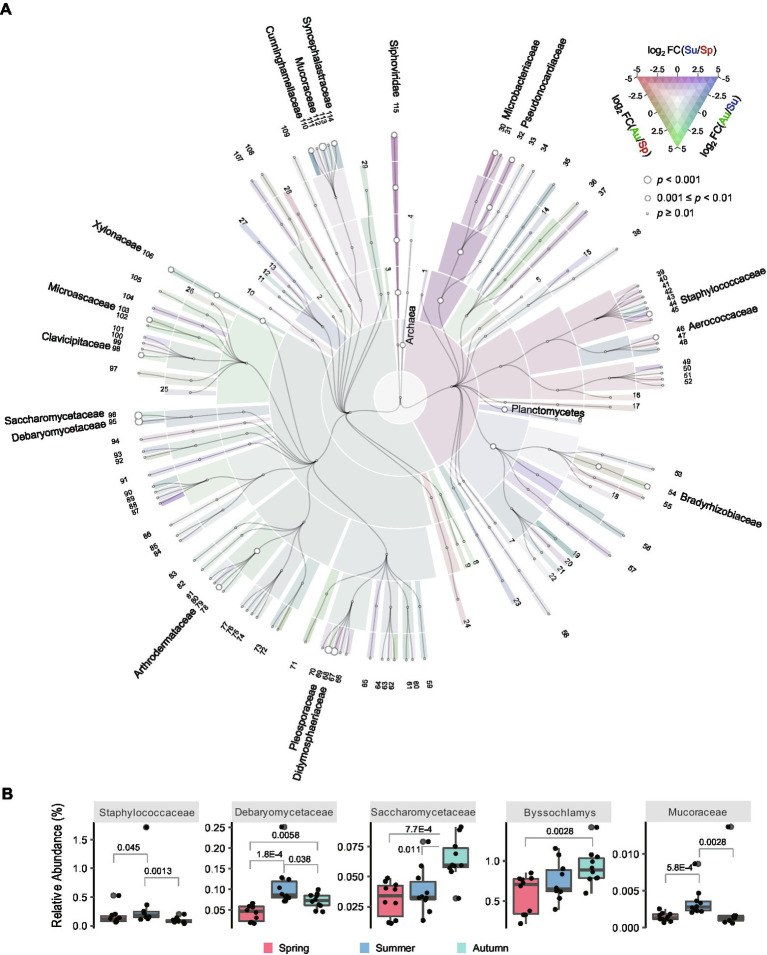
Taxonomic abundances of the black *Daqu* microbiota across seasons. **(A)** Cladogram illustrating abundance of taxa (domain to family). Colors indicate the log2 fold change (FC) between each pair of seasons; circle sizes indicate the minimum *p*-value of the three comparisons by pairwise Mann–Whitney U test. Names of the taxa are indicated if their abundance differences were observed (*p*-value < 0.01) in any comparison. Information of the taxa with the labeled numbers are shown in Supplementary Data 3. **(B)** Boxplots showing the abundance of the differential taxa. The boxes mark the first and third quantile and the lines inside the boxes mark the median; the whiskers extend from the ends of the inter-quartile range (IQR) to the furthest observations within the 1.5 times the IQR. Individual data points are overlaid as dots. The *p*-values are indicated if they are <0.05.

To explore the taxonomic abundance differences of the microbial communities among the three seasons, fold change (FC) values of the abundances were computed pairwisely between two seasons and Mann–Whitney U test was performed for *p*-value calculation (Supplementary Data 3). The results of black *Daqu* are visualized in Figure 3; Supplementary Figure 10. The abundance of Archaea (summer/spring, FC = 1.52, *p*-value = 0.0073) was higher in summer than that in spring. Bacteria in the phylum Planctomycetes (summer/spring, FC = 2.64, *p*-value = 0.0010; autumn/summer, FC = 0.402, *p*-value = 0.0036), as well as the families Microbacteriaceae (autumn/summer, FC = 0.349, *p*-value = 0.0091), Pseudonocardiaceae (autumn/summer, FC = 0.342, *p*-value = 0.0046), Staphylococcaceae (summer/spring, FC = 2.17, *p*-value = 0.045; autumn/summer, FC = 0.272, *p*-value = 0.0013) and Aerococcaceae (summer/spring, FC = 1.26, *p*-value = 0.0073; autumn/summer, FC = 0.623, *p*-value = 0.038) were more abundant in summer. *Saccharomonospora* and *Saccharomonospora* are two genera in the family Pseudonocardiaceae. *Saccharomonospora* has been reported as one of the main bacteria in *Daqu* (Shang et al., 2022), and can hydrolyze phenolic compounds into a non-toxic form and reduce phytotoxicity (Shivlata and Tulasi, 2015). Previous studies have demonstrated that *Saccharopolyspora* is the third largest dominant microbial clusters in a central black component of *Daqu* (Li et al., 2014), and is essential for generating flavoring substances in the downstream process of liquor production (Gan et al., 2019). *Saccharopolyspora* sp. is known to produce a thermostable alpha-amylase which helps to hydrolyze starch (Chakraborty et al., 2011). *Staphylococcus* in the family Staphylococcaceae has been reported as a biomarker in black *Daqu* in a previous metagenomics study (Wang et al., 2021), and shows the potential to participate in butane-2,3-diol metabolism (Yang et al., 2021).

Yeasts in the family Saccharomycetaceae (autumn/spring, FC = 2.08, *p*-value = 0.00077; autumn/summer, FC = 1.67, *p*-value = 0.011) and Debaryomycetaceae (summer/spring, FC = 2.57, *p*-value = 0.00018; autumn/spring, FC = 1.698959872, *p*-value = 0.0058; autumn/summer, FC = 0.661920859, *p*-value = 0.038) were more abundant in autumn and summer, respectively. *Saccharomyces* in the family Saccharomycetaceae is responsible for the production of ethanol, while non-*Saccharomyces* yeasts are responsible for the production of different kinds of esters as flavoring compounds (Zou et al., 2018). *Debaryomyces* sp. has been reported promoting the generation of ethyl hexanoate in liquor producing environment (Tao et al., 2013).

Fungi in Cunninghamellaceae (summer/spring, FC = 5.50, *p*-value = 0.0046; autumn/summer, FC = 0.505, *p*-value = 0.0022), Mucoraceae (summer/spring, FC = 2.45, *p*-value = 0.00058; autumn/summer, FC = 0.694, *p*-value = 0.0028), and Syncephalastraceae (summer/spring, FC = 2.78, *p*-value = 0.0013; autumn/summer, FC = 0.811, *p*-value = 0.0028) were more abundant in summer, while fungi in *Byssochlamys* (autumn/spring, FC = 1.59, *p*-value = 0.0028) were more abundant in autumn. Molds in the family Mucoraceae are known to be strong amylase producers in amylolytic Asian fermentation starters, and have been isolated and identified in *Daqu* in a previous study (Zheng, 2015). *Byssochlamys* is potentially involved in flavor-relevant pathways like biosynthesis of guaiacol (Yang et al., 2021). We also found other fungi with significant abundance differences across seasons, but their roles in fermentation and liquor production await further investigation. Among all the 17 families showed significant difference among seasons in black *Daqu*, 10 were the most abundant in summer and 7 were the most abundant in autumn, indicating that black *Daqu* produced in the two seasons can contain more microbes for fermentation.

Taxonomic abundance differences among the three seasons were also explored for white and yellow *Daqu*. Only a few taxa were significantly changed across seasons in white *Daqu* (Supplementary Figure 11), indicating that the microbial community structure of white *Daqu* was more stable across seasons. Differential taxa of yellow *Daqu* were hardly overlapped with those of black *Daqu* (Supplementary Figures 12; 13). The results demonstrated the distinct differences in seasonal microbial variation across the three types of *Daqu*.

### 3.3. Quantification of key microbial enzymes related to saccharification process

**Figure 4:**
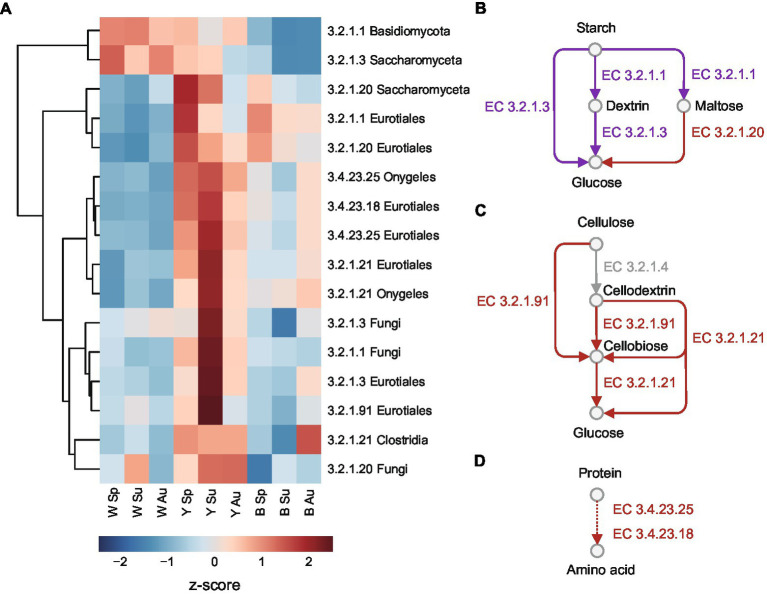
Key enzymes related to saccharification process in *Daqu*. **(A)** Heatmap in relative abundance of the enzymes of different taxa. Abundances are normalized to z-scores, which are in the units of standard deviation from the mean. **(B–D)** Enzymes related to the hydrolysis pathway of **(B)** starch to glucose, **(C)** cellulose to glucose, and **(D)** protein to amino acids. The red arrows are enzymes more abundant in yellow *Daqu*, while the purple arrows are enzymes with divergent abundance patterns among taxa. The one in grey was not detected in this study.

Based on previous studies on the saccharification process of Chinese liquor brewing (Gan et al., 2019; Fan et al., 2021; Xia et al., 2022), we selected the key enzymes involving in the degradation of biomacromolecules in raw materials, including starches, celluloses, and proteins (Figure 4). Alpha-amylases (EC 3.2.1.1) can break large starches into dextrin and a small amount of maltose during the fermentation process. Alpha-glucosidases (EC 3.2.1.20) and glucoamylases (EC 3.2.1.3) can release glycose from maltose and dextrin. Cellulases are a group of enzymes that can degrade cellulose into glucose, including cellulose 1,4-beta-cellobiosidases (EC:3.2.1.91) breaking cellulose and cellodextrin into cellobiose, and beta-glucosidase (EC 3.2.1.21) releasing glycose from cellodextrin and cellobiose. Proteases like saccharopepsin (EC 3.4.23.25) and aspergillopepsin (EC 3.4.23.18) can carry out protein hydrolysis, which can provide various amino acids as nitrogen sources for the growth of microbes.

The quantified microbial enzymes among the key enzymes were clustered using their abundance patterns measured by quantitative metaproteomics (Figure 4A; Supplementary Figure 14; Supplementary Data 4), where the proteins were annotated using eggNOG (Huerta-Cepas et al., 2019). Except that alpha-amylases of Basidiomycota and glucoamylase of Saccharomyceta were more abundant in white *Daqu*, most of the enzymes were more abundant in yellow *Daqu*. Alpha-amylases and alpha-glucosidases of Eurotiales, as well as alpha-glucosidases of Saccharomyceta were more abundant in spring compared to summer and autumn in yellow and black *Daqu*. The cellulases and proteases of Eurotiales and Onygeles, as well as alpha-amylases, glucoamylases and alpha-glucosidases of other fungi were more abundant in summer yellow *Daqu*. The results suggest that yellow *Daqu* in summer probably has higher saccharifying power for raw material degradation.

### 3.4. Functional characteristics of altered microbial proteins across seasons

**Figure 5:**
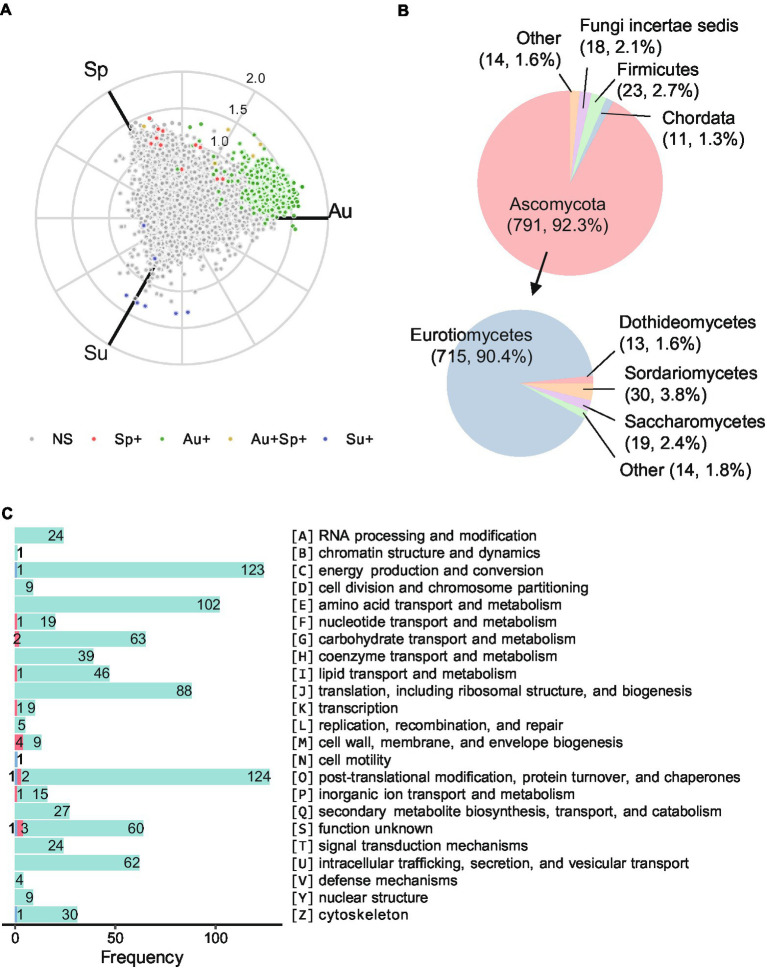
Functional annotations of the differential proteins in black *Daqu* microbiota across seasons. **(A)** Three-axis polar plot indicating the differential proteins across seasons. Proteins are color-coded for significant higher abundance in different comparisons with fold change (FC) > 2 and adjusted *p*-value < 0.05 by pairwise Mann–Whitney U test. NS: not significant; Sp+: significant higher abundance in spring compared to either of summer and autumn or both; the rest are in the same manner. **(B)** Distribution of taxonomy assigned to the differential proteins. **(C)** Numbers of the differential proteins in each category of clusters of orthologous groups (COG). Proteins more abundant in spring, summer, and autumn are in red, blue, and green, respectively. Sp: spring season; Su: summer season; Au: autumn season.

To explore the functional feature of *Daqu* microbiota across seasons, differential proteins between each pair of seasons were determined by abundance FC (> 2 or <0.5) and Mann–Whitney U test (*p*-value < 0.05 with the Benjamini-Hochberg adjustment). While almost no significantly differential protein was observed in white and yellow *Daqu* across seasons, 1,082 proteins in black *Daqu* exhibited significant differences between at least one season pair (Figure 5A; Supplementary Figure 15). Among them, 1,057 were significantly more abundant in autumn, 11 were significantly more abundant in spring, and 7 were significantly more abundant in summer, compared to at least one of the other two seasons. Moreover, 7 proteins were significantly more abundant in both spring and autumn compared to summer. Among the differential proteins, 857 were annotated with taxonomy information by eggNOG (Supplementary Data 5). The phylum Ascomycota (92.3%) and Firmicutes (2.7%) accounted for large proportions of the differential proteins, and the differential proteins in Ascomycota mainly belonged to the classes Eurotiomycetes (90.4%), Sordariomycetes (3.8%), and Saccharomycetes (2.4%; Figure 5B). The differential proteins were annotated into 23 categories of clusters of orthologous groups (COG; Figure 5C; Supplementary Figure 15). The top three categories were O (post-translational modification, protein turnover, chaperones), C (energy production and conversion), and E (amino acid transport and metabolism).

**Figure 6:**
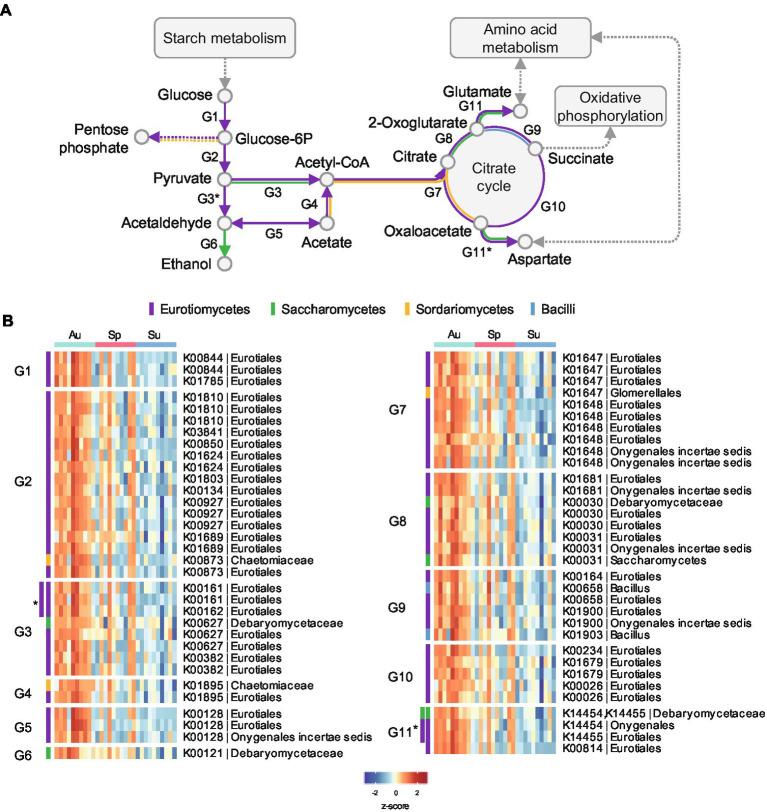
Differential proteins related to the carbohydrate metabolism pathway in black *Daqu* microbiota across seasons. **(A)** Pathway diagram of carbohydrate metabolism. **(B)** Heatmap of the significantly changed microbial proteins. Abundances are normalized to *z*-scores. Proteins are grouped by their related metabolic steps (G1, G2, etc.) in **(A)**. Proteins in the subgroup G3* are related to the metabolic steps G3 and G3*, while other proteins in G3 are not related to the metabolic step G3*; the rest are in the same manner. Proteins are color-coded for different taxa.

The differential proteins were mapped to metabolic pathways in the Kyoto Encyclopedia of Genes and Genomes (KEGG) database (Kanehisa et al., 2017) and pathway enrichment analysis was performed (Supplementary Figure 16; Supplementary Data 6). The enriched pathways in autumn black *Daqu* microbiota were mainly involved in carbon metabolism and amino acid metabolism, which is consistent with the COG annotation result. As shown in Figure 6; Supplementary Data 7, enzymes involving glycolysis and the citrate cycle of Eurotiomycetes were more abundant in autumn black *Daqu* to consume glucose released from starch and cellulose degradation. Alcohol dehydrogenases of Saccharomycetes were elevated to produce ethanol. Some enzymes involving the citrate cycle were also increased in Sordariomycetes and Bacilli. The results suggest that autumn black *Daqu* has better potential for carbohydrate degradation and flavor compounds metabolism than the other seasons.

## 4. Conclusion

We used DIA-based quantitative metaproteomics to reveal the microbial profiles of a cohort of different types of *Daqu* across seasons. Taxonomic composition varied in the microbial communities of different types of *Daqu*, where the proportion of lactic acid bacteria and microbial diversity reduced in yellow and black *Daqu* compared to the under-fermented white *Daqu*. Higher abundance of key enzymes such as alpha-amylases, cellulases, and proteases suggested the higher saccharifying power for raw material degradation of microbes in yellow *Daqu*, which is indeed the main part of the starter used for sauce-flavor liquor production. Seasonal changes of the microbial communities of different types of *Daqu* were also explored. While the under-fermented white *Daqu* had relatively stable microbial community structure, abundances of many taxa were significantly changed across seasons in yellow and black *Daqu*. In addition, abundance of microbial proteins exhibited considerable variation in the over-fermented black *Daqu*, where the abundance of proteins involved in carbohydrate and amino acid metabolism elevated in autumn. The unique microbial characteristics of different types of *Daqu* and their seasonal features are anticipated to guide the maintenance of yield, quality, and flavor in liquor production. Further studies are needed to profile the *Daqu* microbial communities in time series of the fermentation process and with specific environmental factors, towards a deeper understanding of the traditional *Baijiu* brewing technique. We also expect that the DIA-based quantitative metaproteomics method can boost the study of other, diverse fermenting foods.

## Data availability statement

All raw MS data and search results, as well as the protein sequence database generated in this study have been deposited to the ProteomeXchange via the iProX (Ma et al., 2019) partner repository with accession numbers PXD036834 (http://proteomecentral.proteomexchange.org/cgi/GetDataset?ID=PXD036834) or IPX0005077000 (https://www.iprox.cn/page/project.html?id= IPX0005077000). The raw metagenomic sequence data have been deposited to the Genome Sequence Archive (Chen et al., 2021) in National Genomics Data Center (CNCB-NGDC Members and Partners, 2022), China National Center for Bioinformation / Beijing Institute of Genomics, Chinese Academy of Sciences with accession number CRA008200 (https://ngdc.cncb.ac.cn/gsa/browse/CRA00820). Custom scripts for data processing and visualization in this paper are available at Github (https://github.com/lmsac/metaproteomics-utilities).

## Author contributions

JZha performed the majority of the experiments, analyzed the data, and wrote the first draft of the manuscript. YY analyzed the data and revised the manuscript. LC and XL collected the *Daqu* sample. JZhe, DL, and ZF assisted in the microbial and proteomic experiments. CS analyzed the MS data. VM and YL performed the whole genome sequencing data analysis. SY provided the MS resources for data collection. FY, LW, and LQ designed the study. LW and LQ supervised all aspects of the study and finalized the manuscript. All authors contributed to the article and approved the submitted version.

## Funding

This work was supported by the National Natural Science Foundation of China (22022401 and 22074022).

## Conflict of interest

LC, XL, FY, and LW are employed by Kweichow Moutai Group. CS is employed by Shanghai Omicsolution Co., Ltd.

The remaining authors declare that the research was conducted in the absence of any commercial or financial relationships that could be construed as a potential conflict of interest.

## Publisher’s note

All claims expressed in this article are solely those of the authors and do not necessarily represent those of their affiliated organizations, or those of the publisher, the editors and the reviewers. Any product that may be evaluated in this article, or claim that may be made by its manufacturer, is not guaranteed or endorsed by the publisher.
