# Supplementary material for: Quantitative metaproteomics reveals composition and metabolism characteristics of microbial communities in Chinese liquor fermentation starters
Source: Front Microbiol. 2023 Jan 9;13:1098268. doi: 10.3389/fmicb.2022.1098268 (PMC9868298; doi:10.3389/fmicb.2022.1098268)
Supplement: Supplementary file 8 [file Data_Sheet_8.pdf]

frontiers

Supplementary Material

1 Supplementary Notes

Performance comparison of different protein extraction methods.

We benchmarked three current mainstream lysates for protein extraction from *Daqu* microbiota, i.e., 1% SDS, 1% SDC, and BPP solution. As shown in **Supplementary Figure 2A**, the BPP method covered 84% of the protein detected by the SDS method, and provided 81% more proteins. The SDS method yielded slightly more proteins than the SDC methods. Similar results were obtained at the peptide level. Therefore, the BPP lysate is more suitable for *Daqu* protein extraction.

We next compared two cell disruption methods, i.e., mechanical grinding and liquid nitrogen grinding. As shown in **Supplementary Figure 2B**, the mechanical grinding method covered more than 87% proteins and 72% peptides detected by the liquid nitrogen grinding method, and additionally detected 575% proteins and 780% peptides. Due to the complex composition of the *Daqu* sample, the plant tissue in the sample could affect the sufficiency of liquid nitrogen grinding. Therefore, mechanical grinding with a tissue grinder is more suitable for extracting microbial proteins from *Daqu*.

As a result, mechanical grinding with the BPP lysate is the optimal method for protein extraction, and was used for the treatment of the 90 individual *Daqu* samples.

2 Supplementary Figures and Tables

2.1 Supplementary Figures

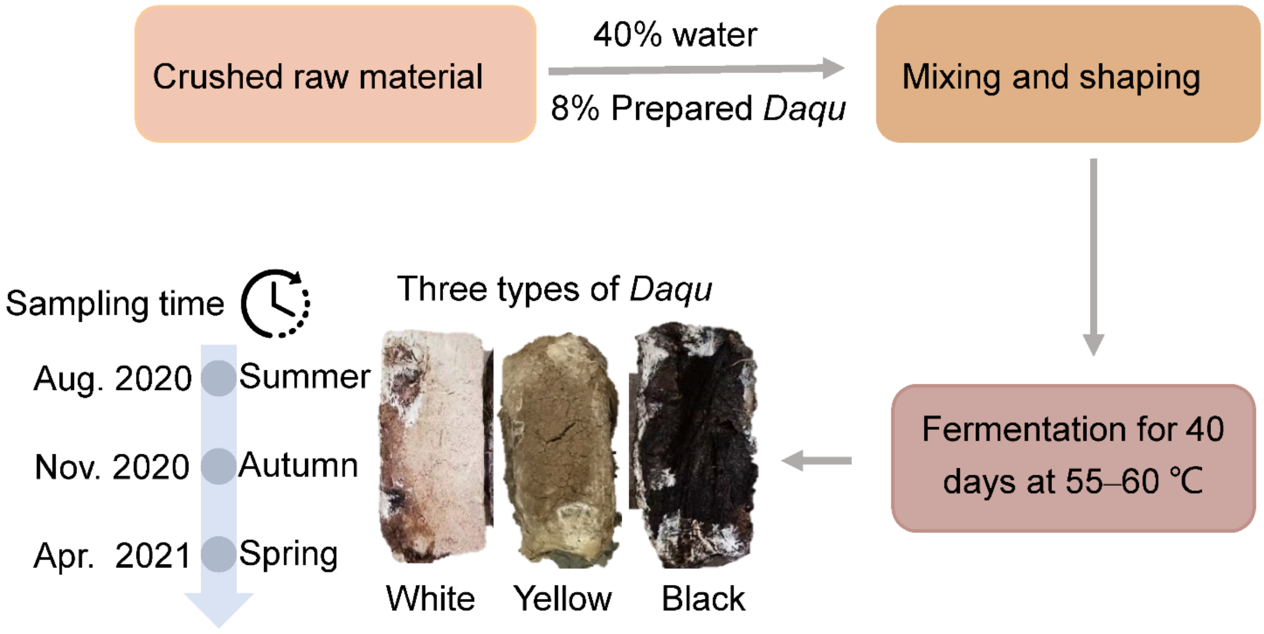

Supplementary Figure 1. Schematic illustration of the production process of three types of *Daqu*. The production process includes two main steps, i.e., shaping and fermenting. First, crushed raw materials, such as wheat, are mixed with 40% water and 8% prepared *Daqu*, and then the mixture is made into bricks. Next, the bricks are piled inside a warehouse and fermented under 55–60 °C for 40 days. Then, the workers dismantle starter brick walls and get immature starter, which can be separated into three types by color, i.e., white starter, yellow starter, and black starter. The immature starters are stored and then mixed for liquor fermentation in actual production. In this study, there batches of immature starters were sampled in summer, autumn, and spring seasons.

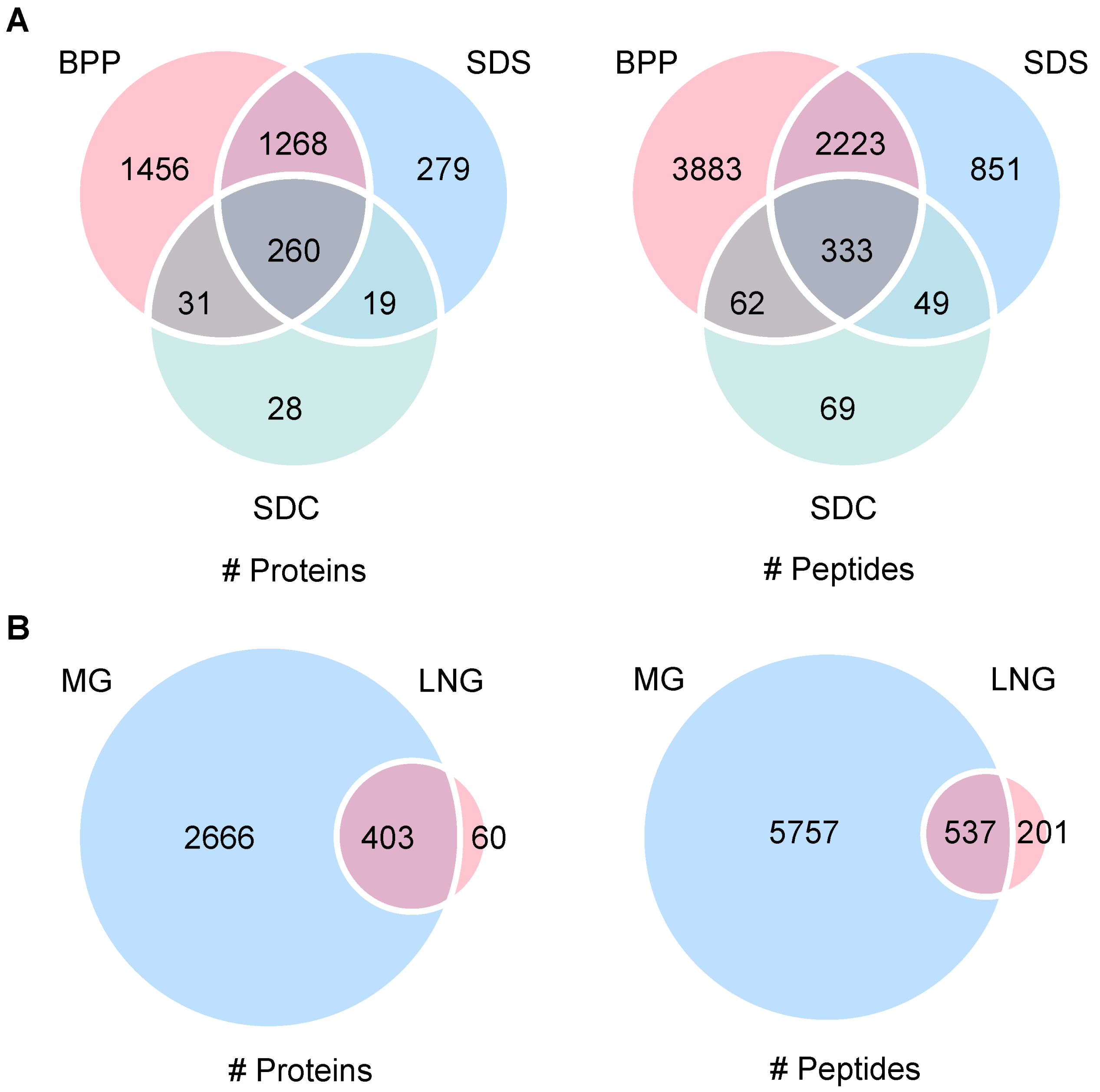

Supplementary Figure 2. Performance comparison of different protein extraction methods. (**A**) Numbers of proteins and peptides detected by LC-MS/MS using different cell lysates. BPP: borax/polyvinylpolypyrrolidone/phenol lysate; SDS: sodium dodecyl sulfate lysate; SDC: sodium deoxycholate lysate. (**B**) Numbers of detected proteins and peptides using different cell disruption methods. MG: mechanical grinding; LNG: liquid nitrogen grinding.

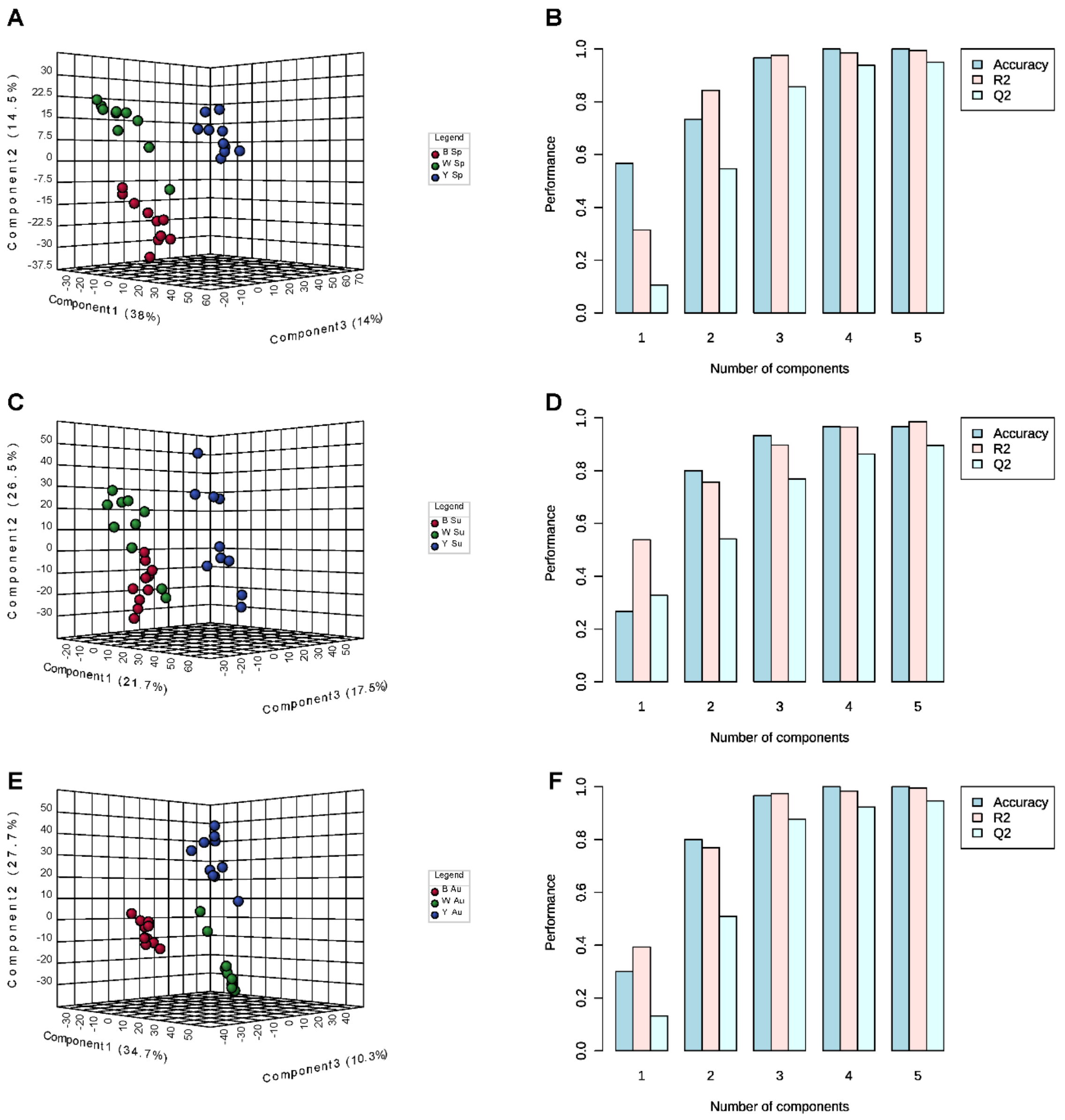

Supplementary Figure 3. PLS-DA results of metaproteome profiles of white, yellow, and black Daqu. (**A–B**) Scores plot (**A**) and performance measures (**B**) of the PLS-DA model of *Daqu* in spring. (**C–D**) Scores plot (**C**) and performance measures (**D**) of the PLS-DA model of *Daqu* in summer. (**E–F**) Scores plot (**E**) and performance measures (**F**) of the PLS-DA model of *Daqu* in autumn. W: white; Y: yellow; B: black; Sp: spring season; Su: summer season; Au: autumn season.

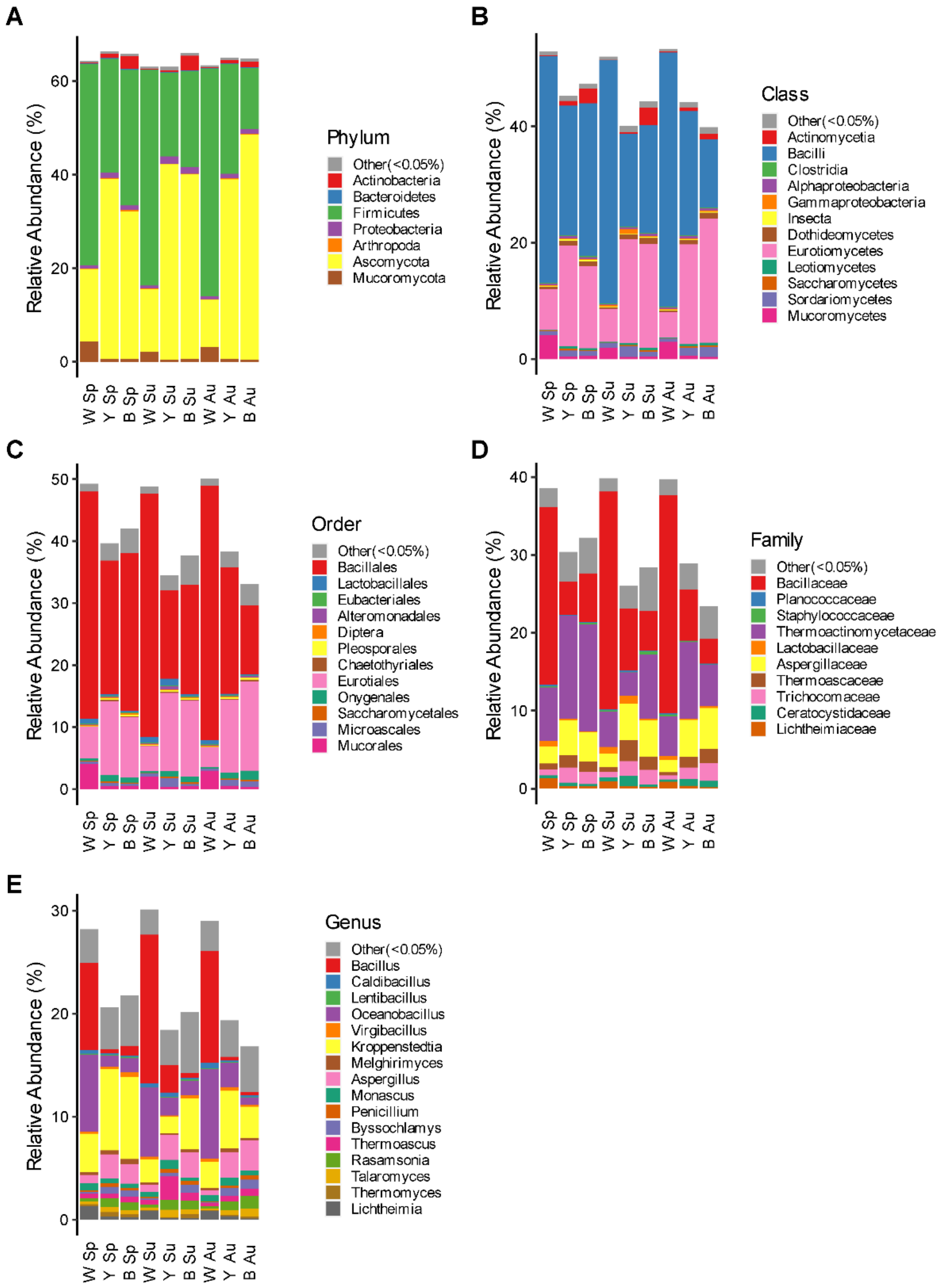

Supplementary Figure 4. Taxonomic profiles based on peptide quantities. (**A**) Relative abundances at the phylum level. (**B**) Relative abundances at the class level. (**C**) Relative abundances at the order level. (**D**) Relative abundances at the family level. (**E**) Relative abundances at the genus level. W: white; Y: yellow; B: black; Sp: spring season; Su: summer season; Au: autumn season.

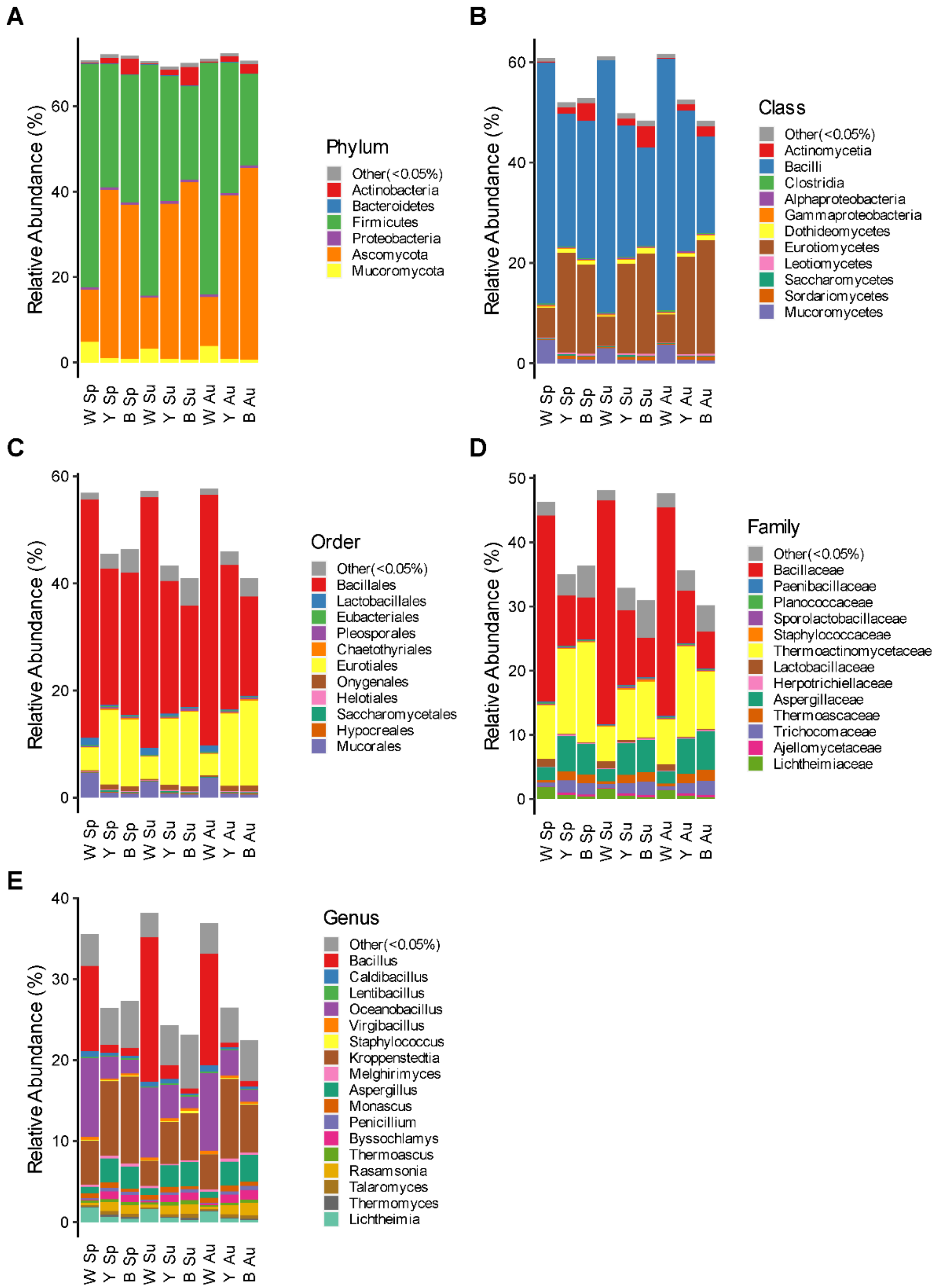

Supplementary Figure 5. Taxonomic profiles based on peptide counts. (**A**) Relative abundances at the phylum level. (**B**) Relative abundances at the class level. (**C**) Relative abundances at the order level. (**D**) Relative abundances at the family level. (**E**) Relative abundances at the genus level. W: white; Y: yellow; B: black; Sp: spring season; Su: summer season; Au: autumn season.

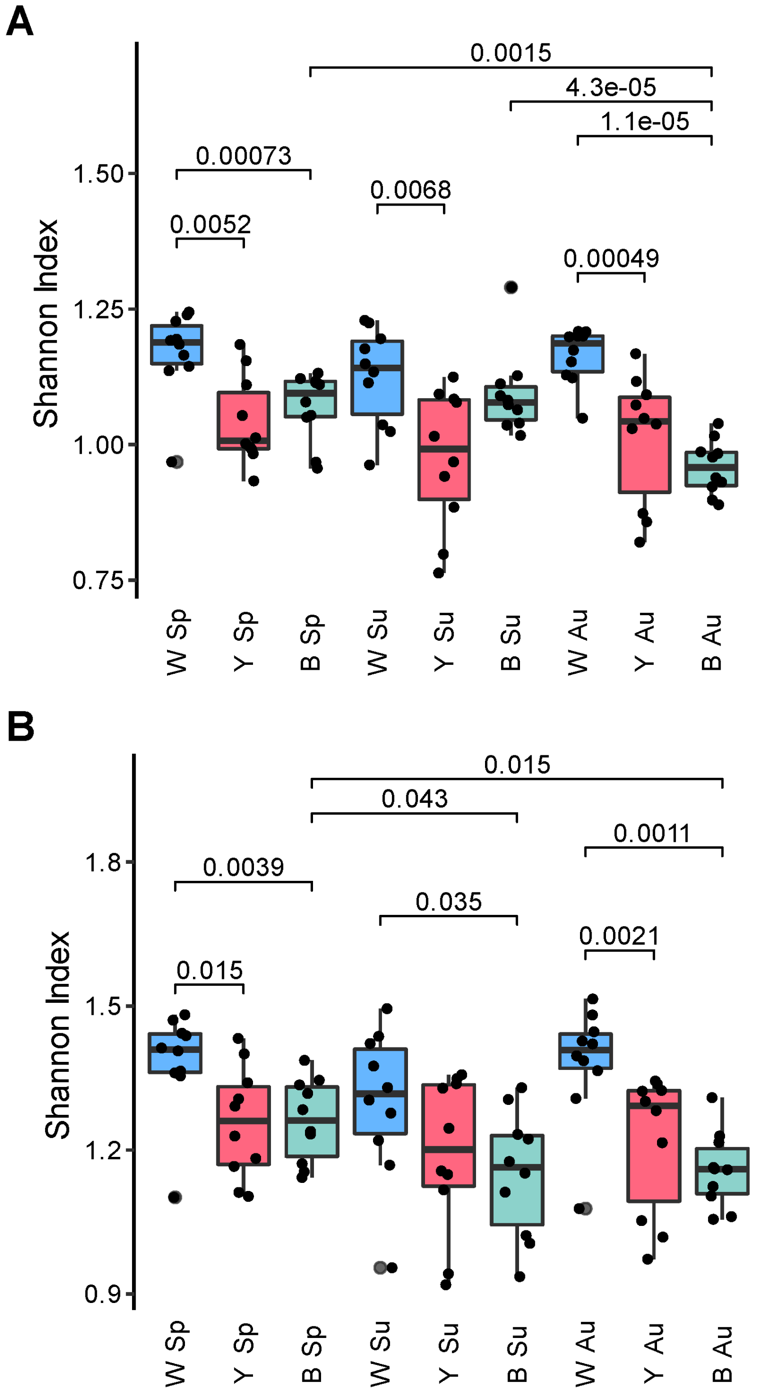

Supplementary Figure 6. Alpha diversity at the genus level. (**A**) Taxonomic abundances calculated based on peptide quantities. (**B**) Taxonomic abundances calculated based on peptide counts. The boxes mark the first and third quantile and the lines inside the boxes mark the median; the whiskers extend from the ends of the inter-quartile range (IQR) to the furthest observations within the 1.5 times the IQR. Individual data points are overlaid as dots. The p-values are indicated if they are < 0.05. W: white; Y: yellow; B: black; Sp: spring season; Su: summer season; Au: autumn season.

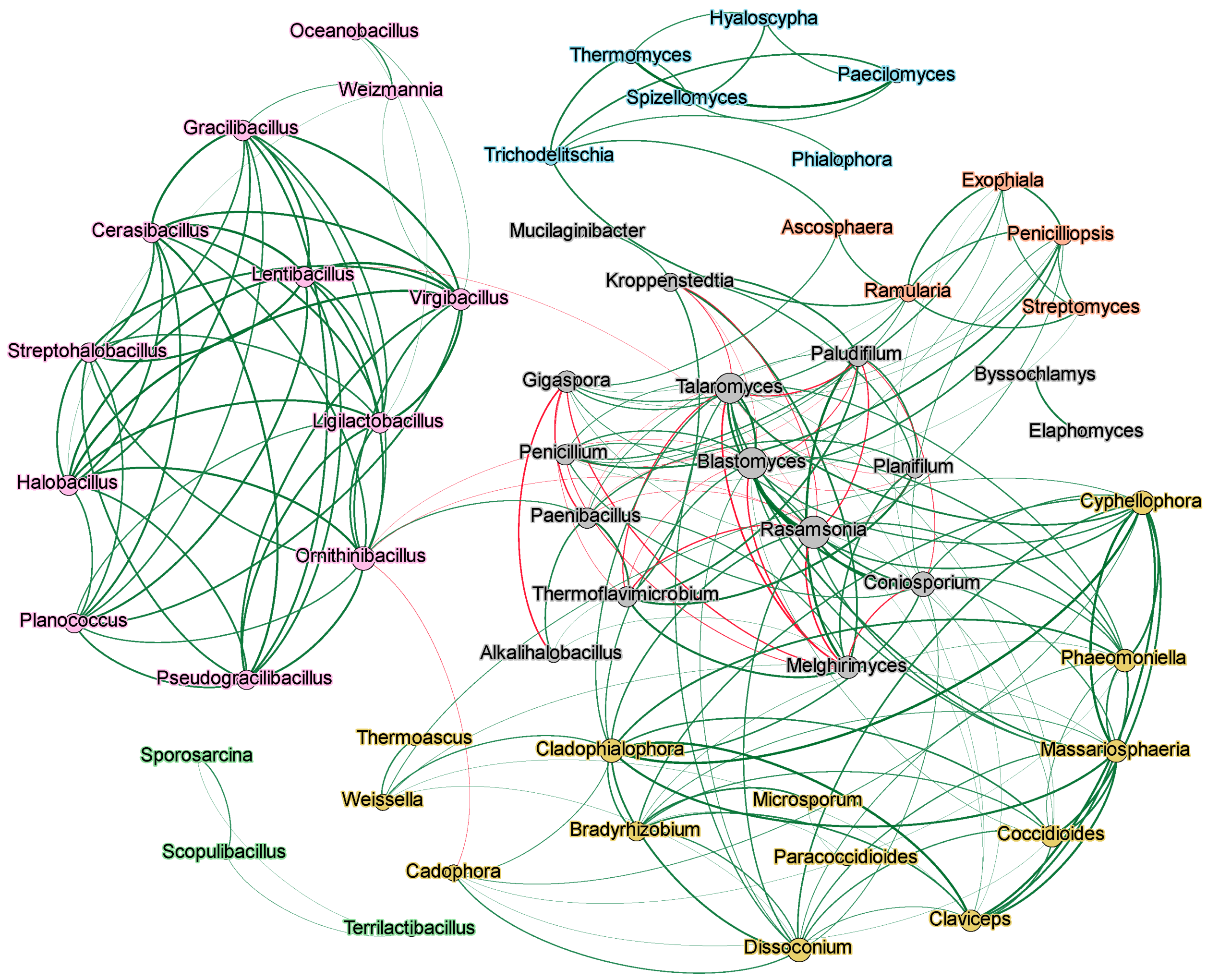

Supplementary Figure 7. Correlation of microbial genera in black *Daqu* samples. Genera with relative abundance > 0.05% are shown as nodes. Color of each edge indicates a negative (red) or positive (green) significant correlation (Spearman's correlation > 0.8, p-value < 0.05). Thickness of each edge is proportional to Spearman's correlation. Size of each genera node is proportional to the number of connections. Nodes are color-coded for different clusters (densely connected subgraphs) identified by random walks, while clusters with <3 nodes are in gray.

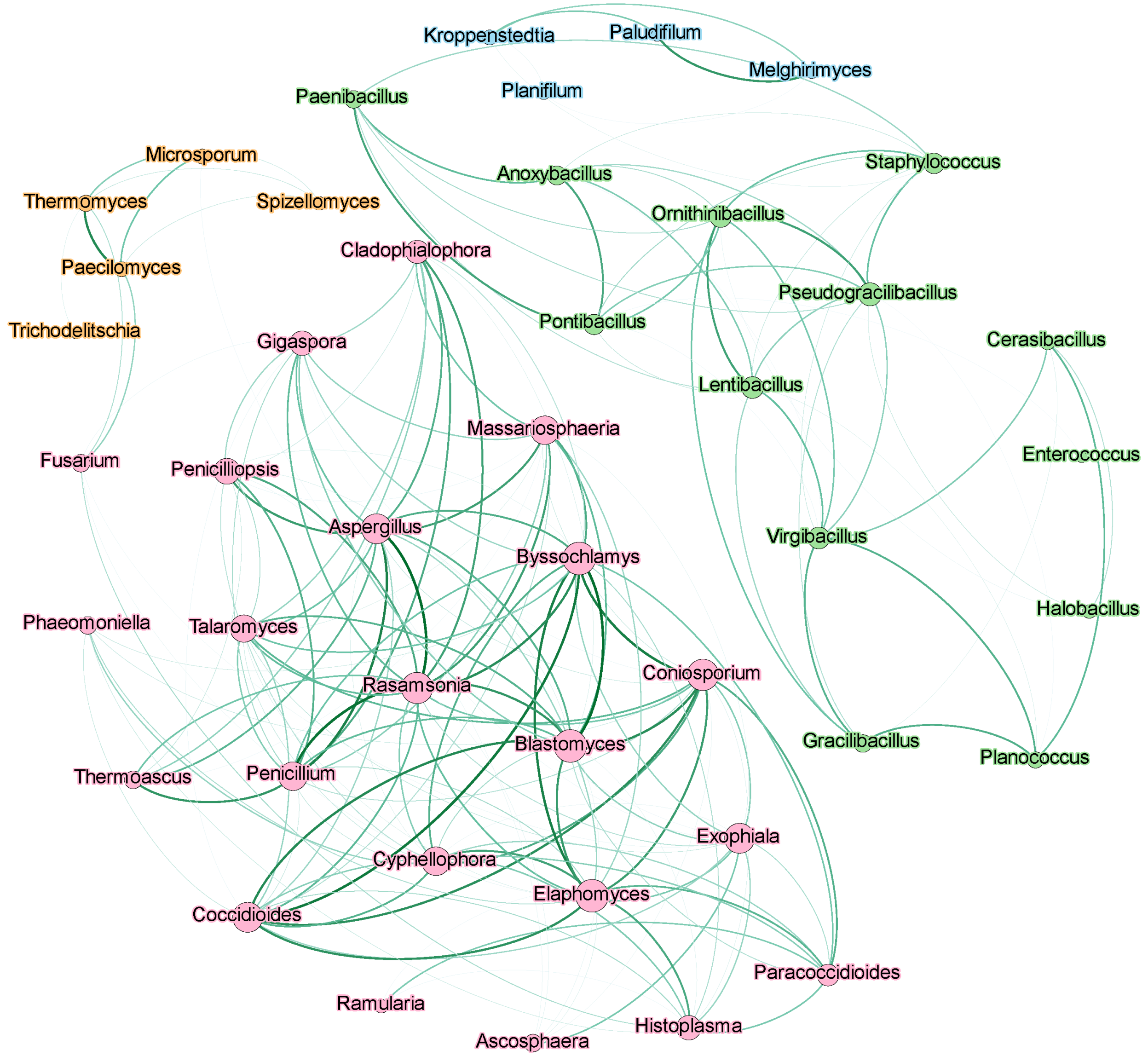

Supplementary Figure 8. Correlation of microbial genera in white *Daqu* samples. Genera with relative abundance > 0.05% are shown as nodes. Color of each edge indicates a negative (red) or positive (green) significant correlation (Spearman's correlation > 0.8, p-value < 0.05 with Bonferroni adjustment). Thickness of each edge is proportional to Spearman's correlation. Size of each genera node is proportional to the number of connections. Nodes are color-coded for different clusters (densely connected subgraphs) identified by random walks.

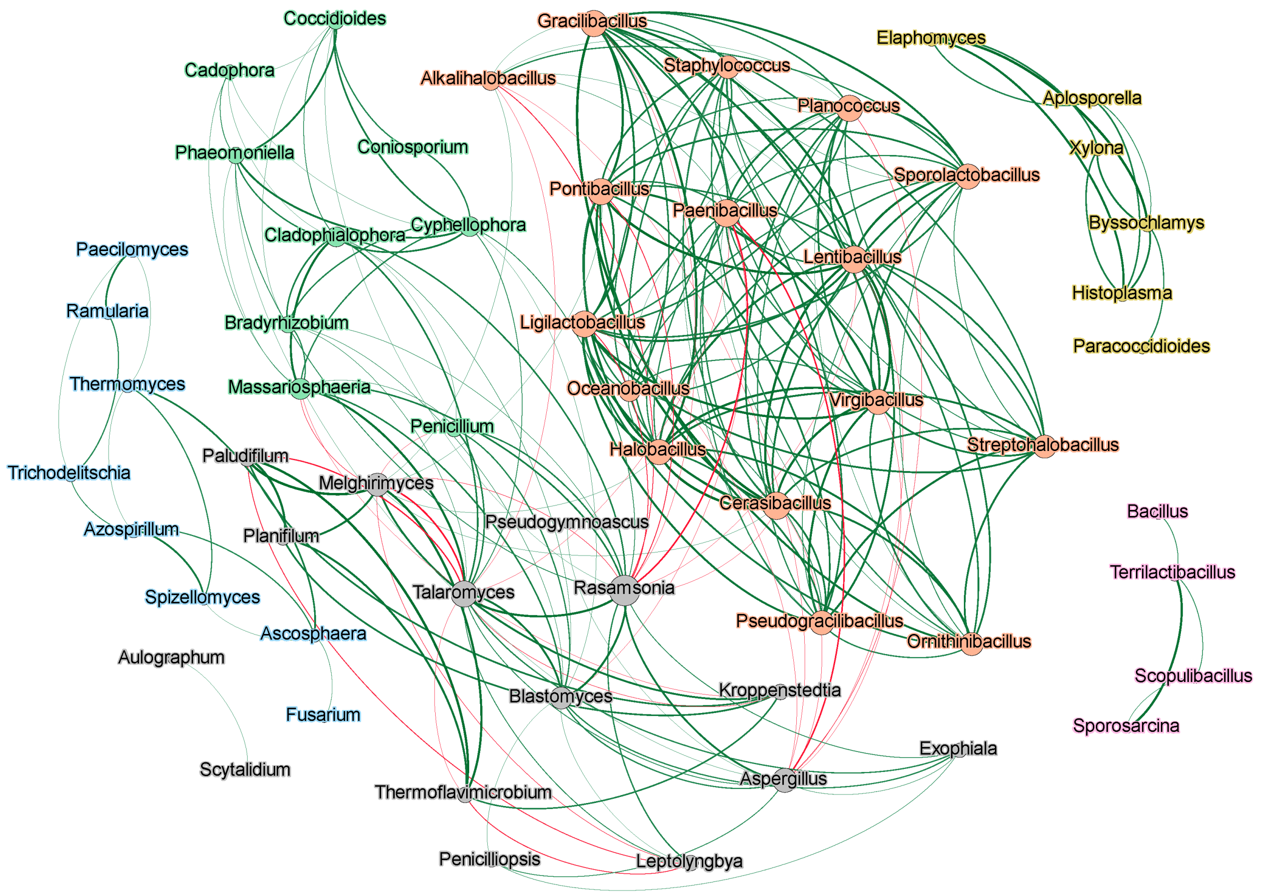

Supplementary Figure 9. Correlation of microbial genera in yellow *Daqu* samples. Genera with relative abundance > 0.05% are shown as nodes. Color of each edge indicates a negative (red) or positive (green) significant correlation (Spearman's correlation > 0.8, p-value < 0.05 with Bonferroni adjustment). Thickness of each edge is proportional to Spearman's correlation. Size of each genera node is proportional to the number of connections. Nodes are color-coded for different clusters (densely connected subgraphs) identified by random walks, while clusters with <3 nodes are in gray.

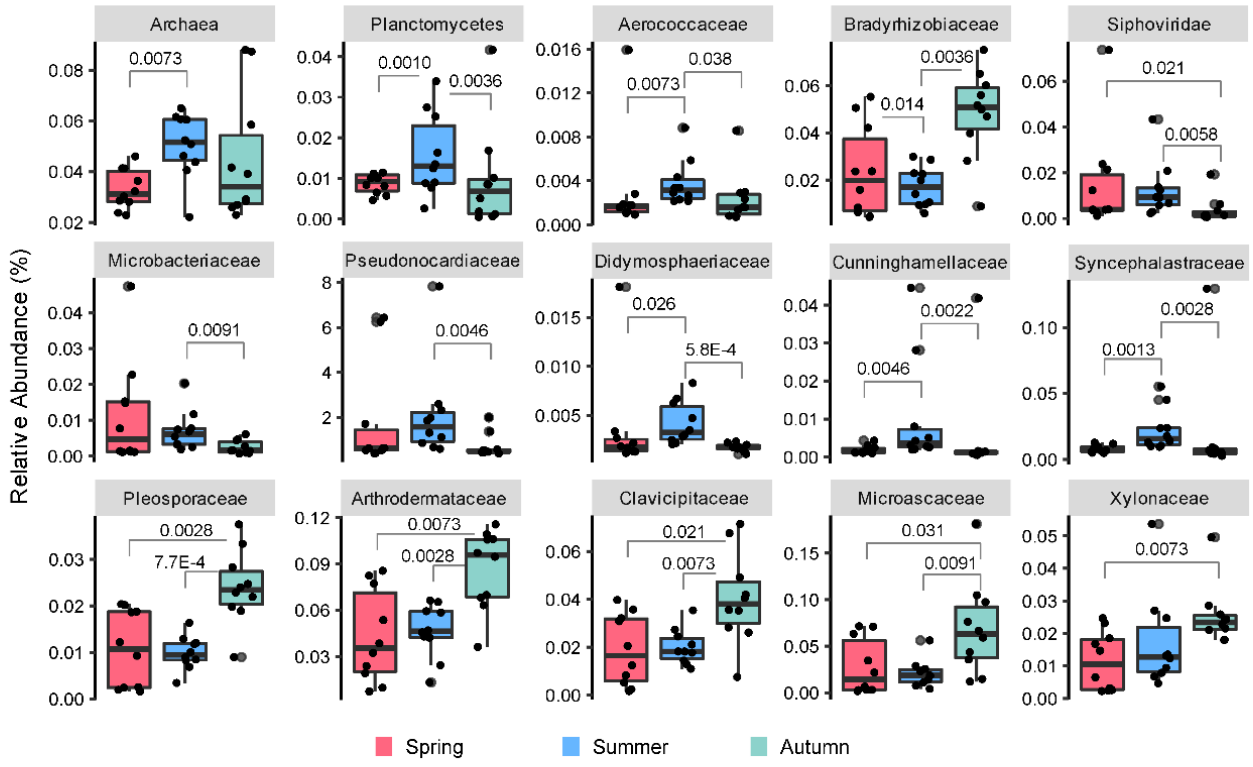

Supplementary Figure 10. Boxplots showing the abundance of the differential taxa in black *Daqu* microbiota across seasons (Continued from **Figure 3B.**). The boxes mark the first and third quantile and the lines inside the boxes mark the median; the whiskers extend from the ends of the inter-quartile range (IQR) to the furthest observations within the 1.5 times the IQR. Individual data points are overlaid as dots. The p-values are indicated if they are < 0.05.

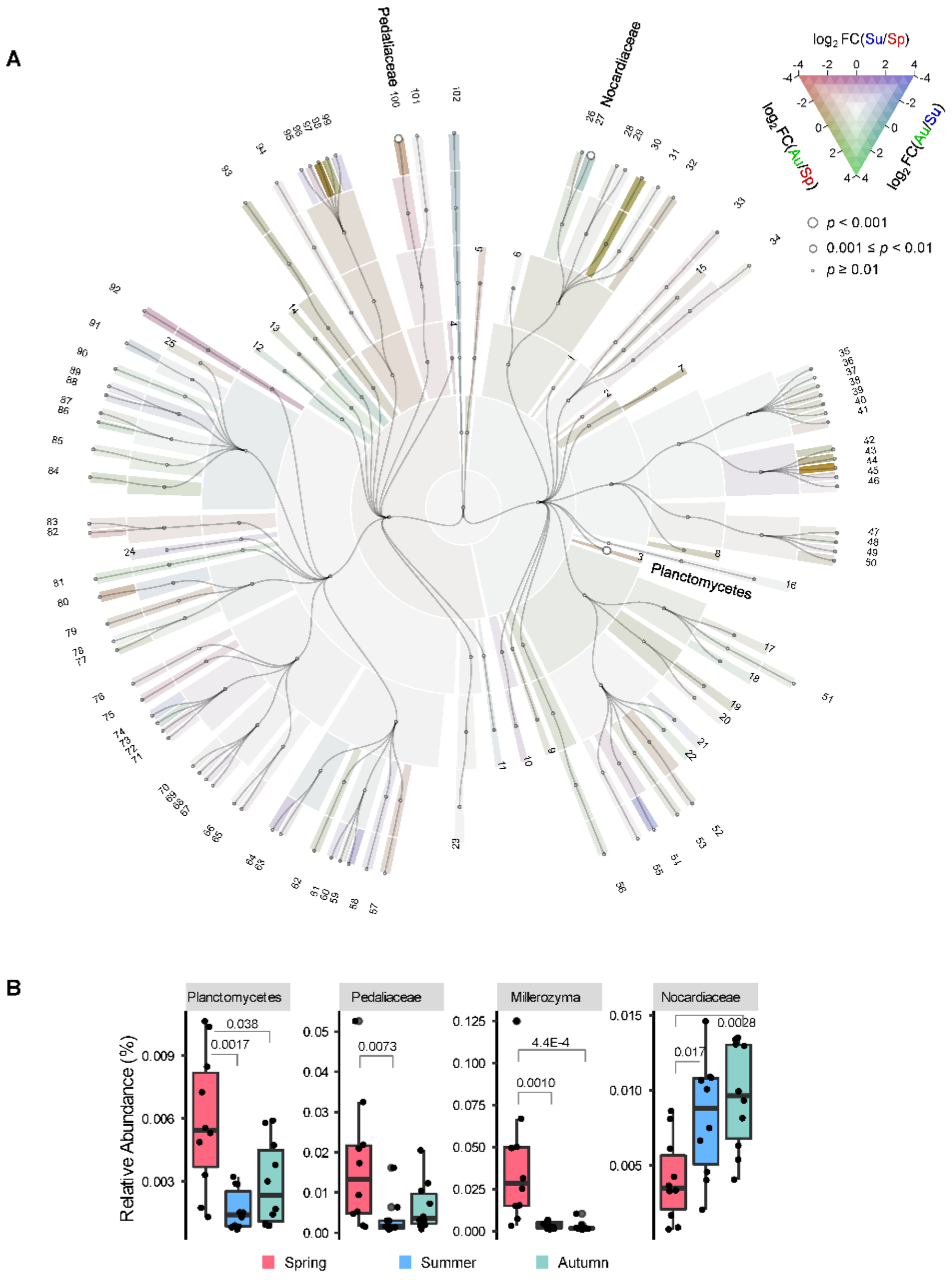

Supplementary Figure 11. Taxonomic abundances of the white *Daqu* microbiota across seasons. (**A**) Cladogram illustrating abundance of taxa (domain to family). Colors indicate the log2 fold change (FC) between each pair of seasons; circle sizes indicate the minimum p-value of the three comparisons by pairwise Mann-Whitney U test. Names of the taxa are indicated if their abundance differences were observed (p-value < 0.01) in any comparison. Information of the taxa with the labeled numbers are shown in **Supplementary Data 3**. (**B**) Boxplots showing the abundance of the differential taxa. The boxes mark the first and third quantile and the lines inside the boxes mark the median; the whiskers extend from the ends of the inter-quartile range (IQR) to the furthest observations within the 1.5 times the IQR. Individual data points are overlaid as dots. The p-values are indicated if they are < 0.05.

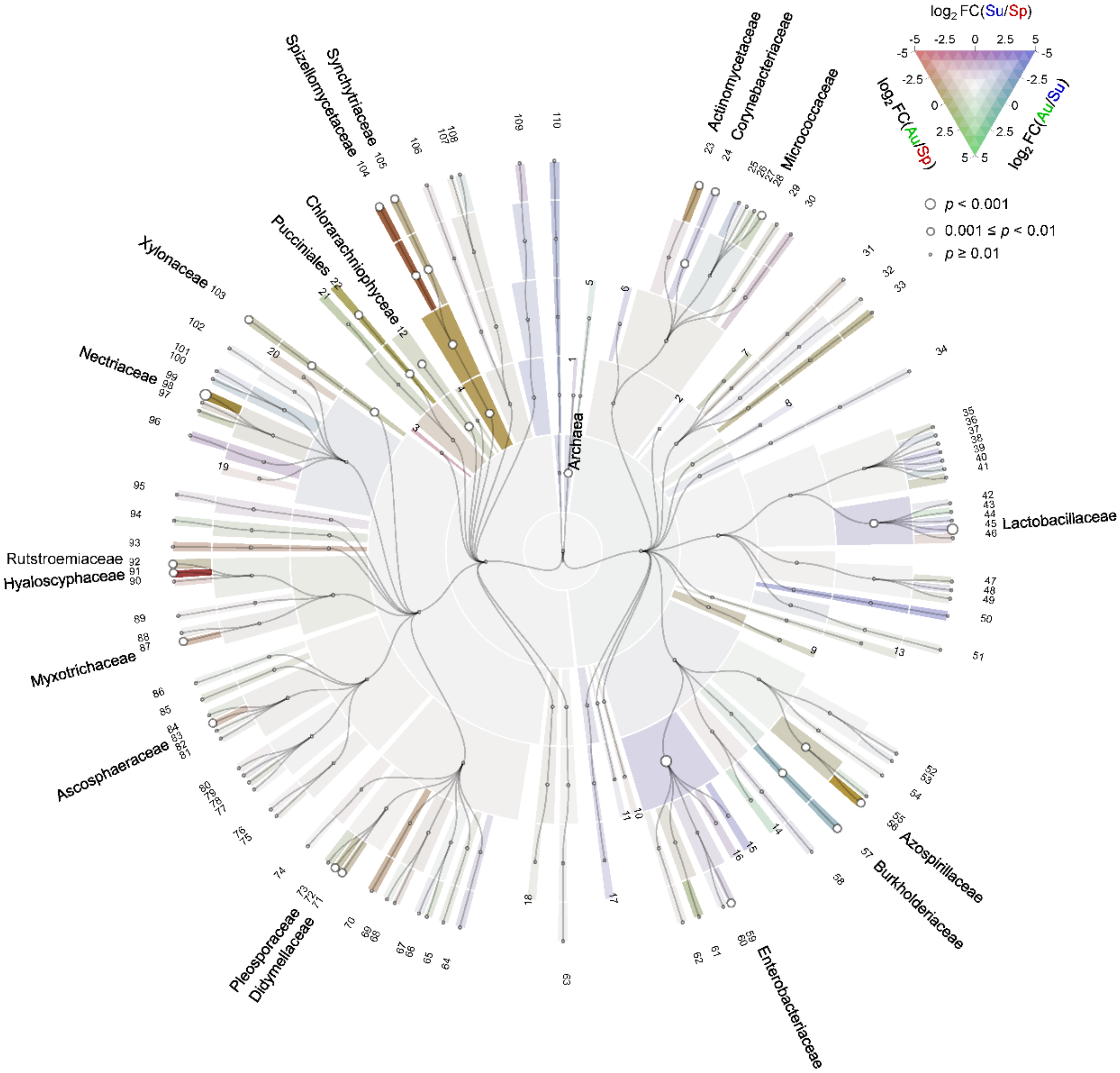

Supplementary Figure 12. Cladogram illustrating abundance of taxa (domain to family) in the yellow *Daqu* microbiota across seasons. Colors indicate the log2 fold change (FC) between each pair of seasons; circle sizes indicate the minimum p-value of the three comparisons by pairwise Mann-Whitney U test. Names of the taxa are indicated if their abundance differences were observed (p-value < 0.01) in any comparison. Information of the taxa with the labeled numbers are shown in **Supplementary Data 3**.

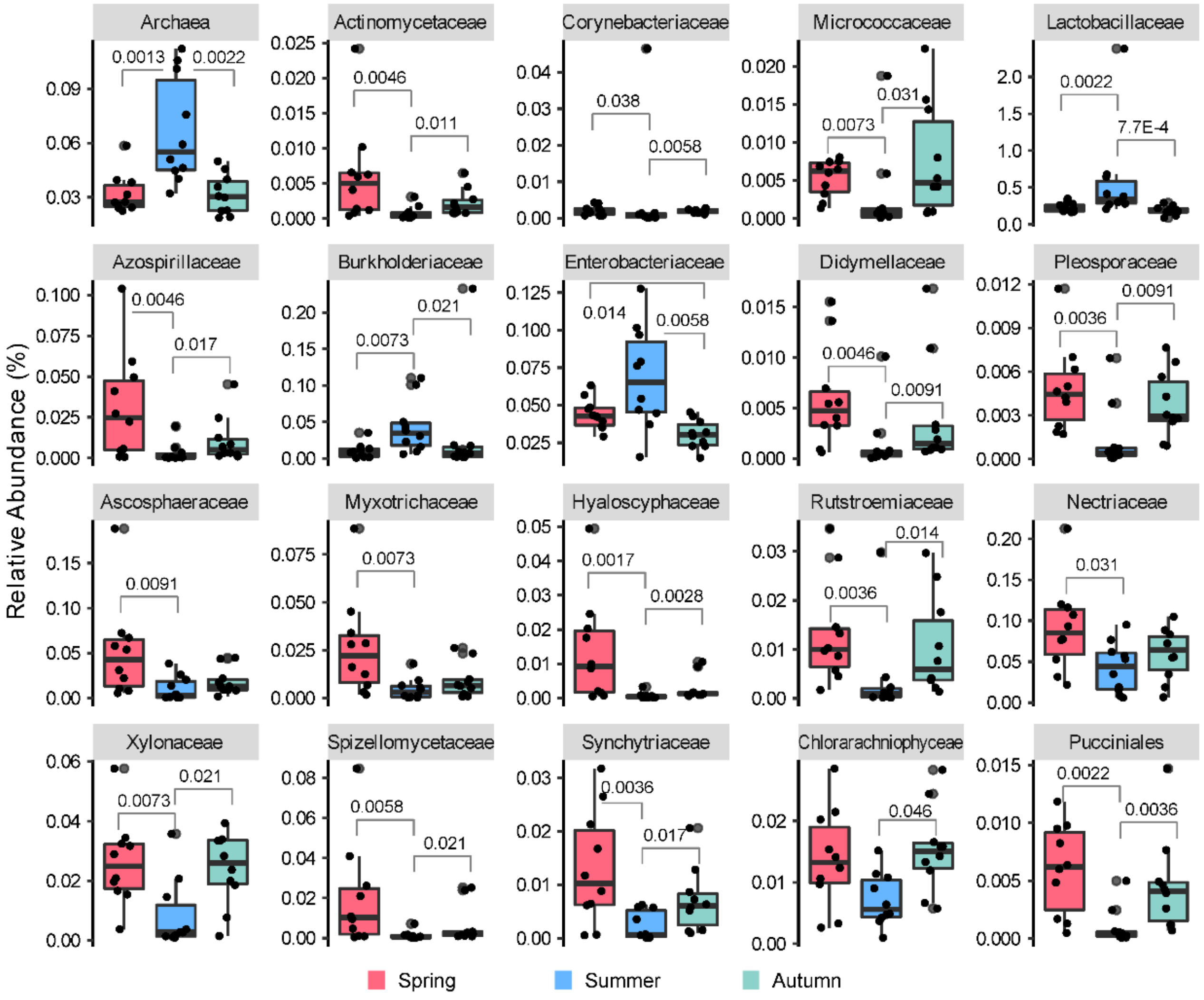

Supplementary Figure 13. Boxplots showing the abundance of the differential taxa in the yellow *Daqu* microbiota across seasons. The boxes mark the first and third quantile and the lines inside the boxes mark the median; the whiskers extend from the ends of the inter-quartile range (IQR) to the furthest observations within the 1.5 times the IQR. Individual data points are overlaid as dots. The p-values are indicated if they are < 0.05.

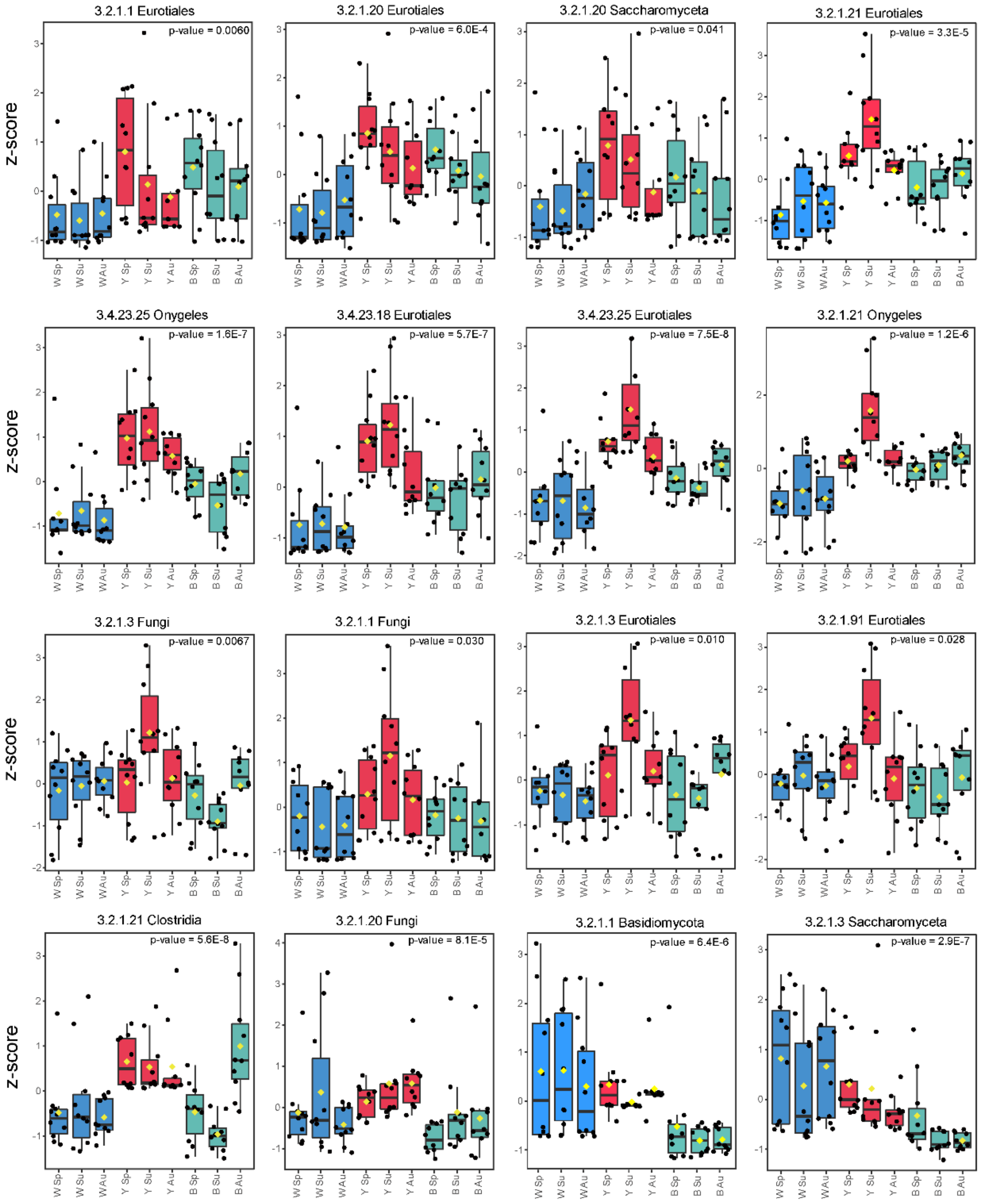

Supplementary Figure 14. Boxplots showing the relative abundance of the key enzymes related to saccharification process in *Daqu*. Abundances are normalized to z-scores, which are in the units of standard deviation from the mean. The boxes mark the first and third quantile and the lines inside the boxes mark the median; the whiskers extend from the ends of the inter-quartile range (IQR) to the furthest observations within the 1.5 times the IQR. Individual data points are overlaid as dots. The p-values by Kruskal-Wallis test are indicated.

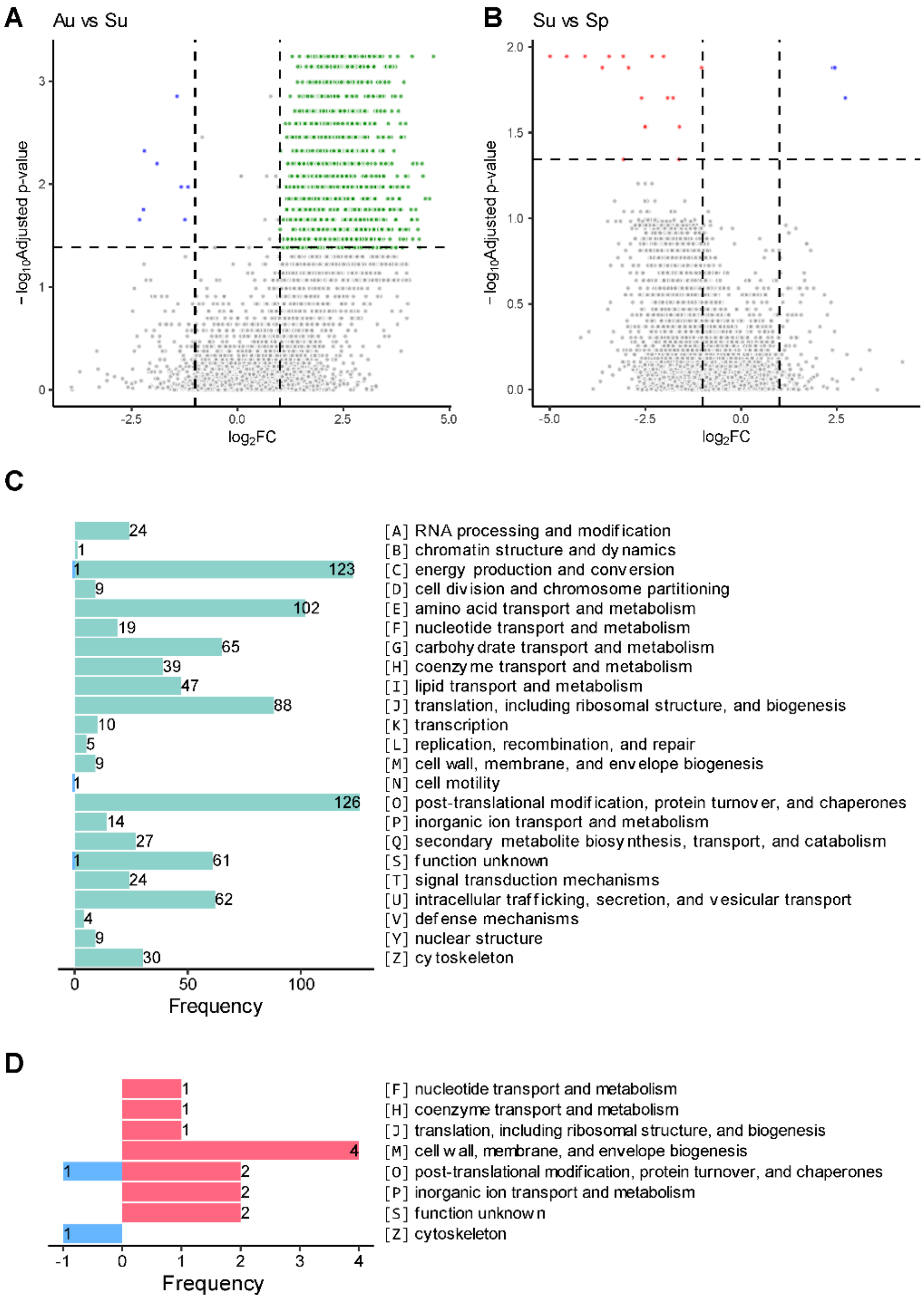

Supplementary Figure 15. Functional annotations of the differential proteins in black *Daqu* microbiota across seasons. (**A-B**) Volcano plots indicating the differential proteins (**A**) between spring and autumn, as well as (**B**) between spring and summer. Proteins are color-coded for significant higher abundance in different comparisons with fold change (FC) > 2 and adjusted p-value < 0.05 by pairwise Mann-Whitney U test. (**C-D**) Numbers of the differential proteins (**C**) between spring and autumn, as well as (**D**) between spring and summer, in each category of clusters of orthologous groups (COG). Proteins more abundant in spring, summer, and autumn are in red, blue, and green, respectively.

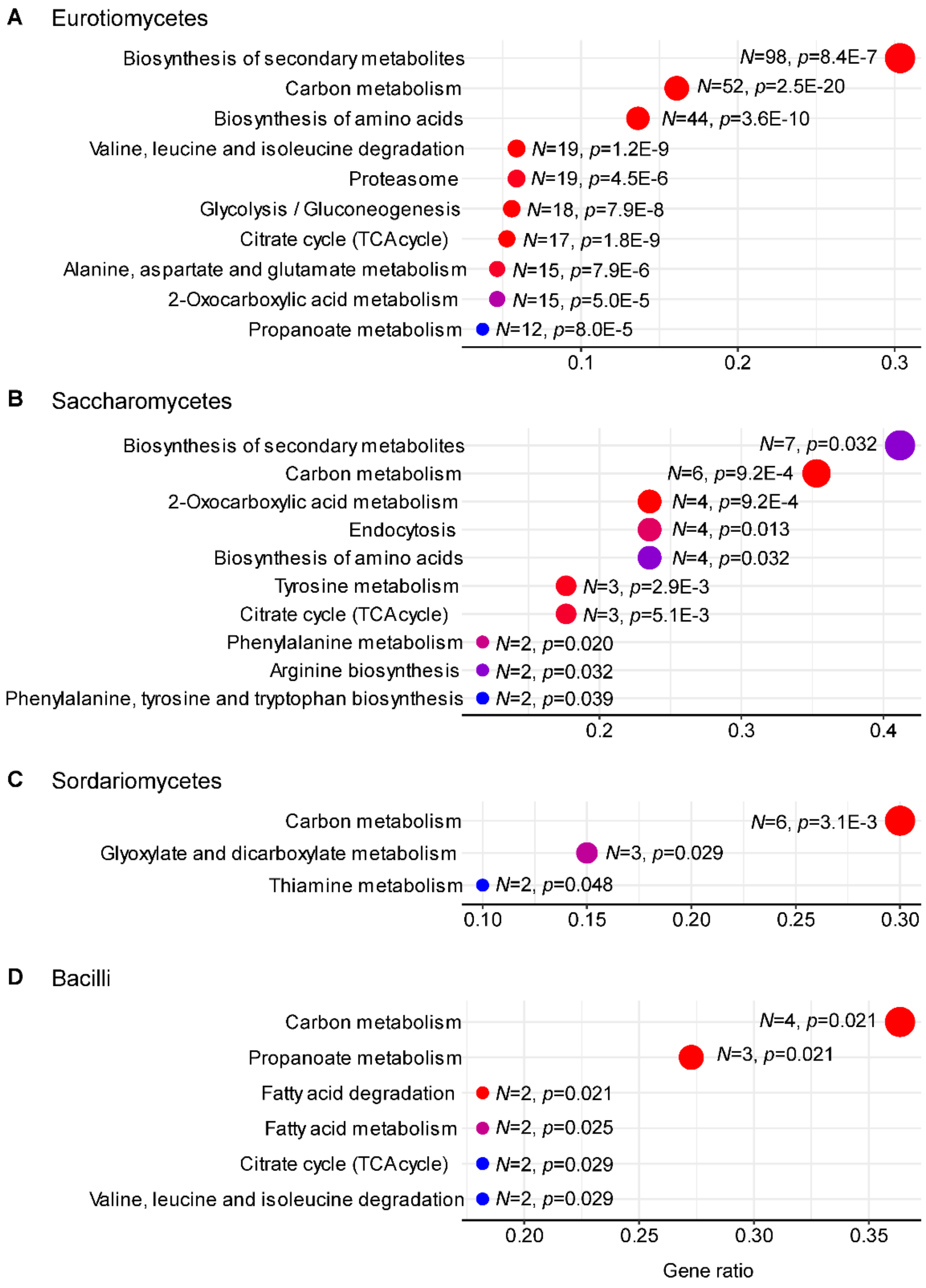

Supplementary Figure 16. Enriched metabolic pathways in black *Daqu* microbiota based on the differential proteins across seasons. (**A**) Enriched pathways of fungi in the class Eurotiomycetes. (**B**) Enriched pathways of fungi in the class Saccharomycetes. (**C**) Enriched pathways of fungi in the class Sordariomycetes. (**D**) Enriched pathways of bacteria in the class Bacilli. Numbers of KO entries (*N*) and adjusted p-values are indicated. Gene ratio: number of the differential genes related to the pathway / number of the total differential genes.

2.2 Supplementary Tables

Supplementary Table 1. Chromatography gradient.

Time	B%
00:00	2%
04:00	5%
45:00	18%
50:00	22%
53:00	32%
56:00	95%
60:00	95%

Supplementary Table 2. DIA variable window settings.

Center	Width	Center	Width
368.5	38	669.5	10
399	25	678.5	10
418	15	688	11
432	15	697.5	10
444.5	12	706.5	10
455	11	716	11
464.5	10	726	11
473.5	10	736.5	12
482.5	10	747.5	12
491	9	758.5	12
499.5	10	769.5	12
508	9	780.5	12
516.5	10	792	13
525	9	804.5	14
533	9	818	15
541.5	10	832.5	16
550	9	848.5	18
558	9	866	19
566.5	10	885.5	22
575.5	10	907	23
584	9	929	23
592	9	951.5	24
600.5	10	974.5	24
609	9	998	25
617.5	10	1024.5	30
626	9	1056	35
634.5	10	1092.5	40
643.5	10	1139	55
652	9	1208	85
660.5	10	1375	251
